# Ionic Liquid-Enabled Drug Delivery Systems: Benefits, Limitations, and Future Perspectives

**DOI:** 10.3390/pharmaceutics18020224

**Published:** 2026-02-10

**Authors:** Daeyeong Lee, Sooa Lim

**Affiliations:** Department of Pharmaceutical Engineering, Hoseo University, Asan-si 31499, Chungnam, Republic of Korea

**Keywords:** ionic liquids, drug delivery system, nanocarriers, transdermal delivery, oral delivery, biocompatibility

## Abstract

ILs have emerged as versatile formulation components in DDS due to their tunable physicochemical properties and ability to modulate biomolecular and interfacial interactions. This review examines IL-enabled DDS strategies across major delivery platforms, including nanocarrier-based systems, microtechnology-assisted devices, and biomacromolecule formulations, with emphasis on formulation design principles rather than administration route. We discuss how ILs enhance API solubility, stability, permeability, and formulation flexibility through API–IL complex formation and controlled membrane interactions and relate mechanistic insights into IL–membrane interactions to both delivery performance and safety via structure–activity relationships. Current limitations, including toxicity concerns, lack of standardized evaluation criteria, scalability challenges, and regulatory ambiguity, are critically assessed. Overall, this review positions ILs as formulation-enabling materials rather than standalone therapeutics and underscores the importance of rational design, standardized assessment, and early regulatory alignment for advancing IL-enabled DDS toward clinical translation.

## 1. Introduction

**Figure 1:**
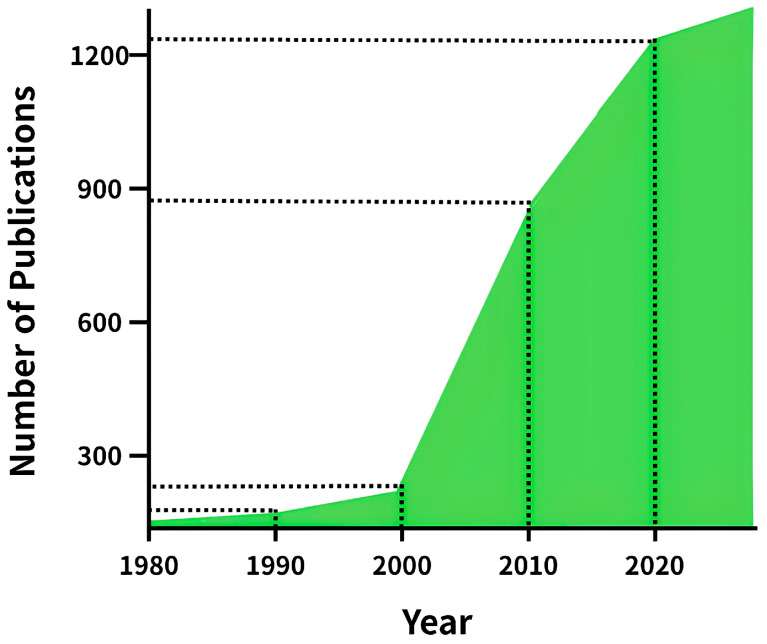
Decades of Development: Cumulative Publications on Ionic Liquids.

Contemporary pharmaceutical development faces persistent challenges associated with the physicochemical properties of active pharmaceutical ingredients (API) and the limitations of conventional drug delivery systems (DDS). Many promising API suffer from poor aqueous solubility and limited bioavailability, while orally administered drugs frequently encounter biological barriers such as low gastrointestinal permeability, variable absorption, and extensive first-pass metabolism, collectively compromising clinical performance [1,2,3,4,5]. To address these issues, innovative DDS approaches—including nanotechnology, microtechnology, and micellar systems—have been extensively investigated to improve API solubility, bioavailability, and targeting efficiency [2,5,6]. Polymer-based DDS have served as foundational materials since the 1980s, and microspheres (1–100 μm) have been widely explored for enhancing drug stability and enabling sustained release [7,8,9]. However, conventional microsphere fabrication frequently relies on organic solvents, which raise environmental and toxicological concerns and may compromise product quality due to residual solvent content [6]. In this context, ionic liquids (ILs) have emerged as promising alternatives capable of mitigating several limitations associated with organic solvent-based DDS [10]. ILs are molten salts composed of asymmetrical bulky organic cations paired with inorganic or organic anions, with melting points often below 100 °C. First reported by Paul Walden in 1914, ILs have been increasingly explored owing to their precisely tunable physicochemical properties, including viscosity, hydrophobicity, solubility, toxicity, and biodegradability [5,10,11,12,13,14]. These attributes have enabled diverse pharmaceutical applications, as demonstrated by choline-based and imidazolium-based ionic liquids, which in selected formulations have been reported to enhance drug solubilization, membrane interactions, and formulation stability in a structure-dependent manner [1,15]. From a pharmaceutical perspective, the biocompatibility, physicochemical tunability, and formulation behavior of API-derived ionic liquids have been comprehensively reviewed, highlighting both their potential and remaining challenges in modern drug delivery systems [16]. Consistent with the exponential PubMed publication growth shown in Figure 1, a WoS-based bibliometric analysis of ILs in drug delivery identified two distinct growth phases—66 publications up to 2013 and 456 publications during 2014–2021, peaking at 83 papers in 2020—while market analysis estimate the global ILs market at USD 53.46 million in 2023 with a projected CAGR of 8.2% (2024–2030), collectively suggesting increasing academic and commercial interest for IL-enabled pharmaceutical technologies (Figure 1) [12,17]. Beyond their role as formulation aids, ILs have been explored as versatile API, antimicrobial agents, stabilizing media for proteins and nucleic acids, and functional components of advanced DDS platforms, including nanoemulsions and microneedle systems [18,19,20,21]. In selected formulations, certain ionic liquids have attracted interest for pharmaceutical applications due to their potential sustainability-related attributes, such as reduced volatility and, in some cases, the use of renewable starting materials; however, these features are not universal and depend strongly on ion selection and formulation context [1]. Accordingly, this review critically analyzes the evolution of IL-based drug delivery systems from a selective, framework-driven perspective, focusing on representative platforms to systematically elucidate structure–function relationships, translational challenges, and key design principles governing their pharmaceutical and biomedical potential.

Here, ILs are strictly defined as salts composed entirely of ions that are liquid at or near room temperature; deep eutectic solvents and eutectic mixtures are therefore excluded as conceptually distinct systems, and this review accordingly adopts a selective, framework-driven approach by focusing on representative IL-enabled platforms that elucidate formulation-driven structure–property–function relationships and translational considerations.

Publication trends (1980–2024) show exponential growth in the cumulative number of papers obtained by searching ‘ionic liquids’ in the PubMed database, reflecting increasing interest from academia and industry.

## 2. Generation of Ionic Liquids

**Figure 2:**
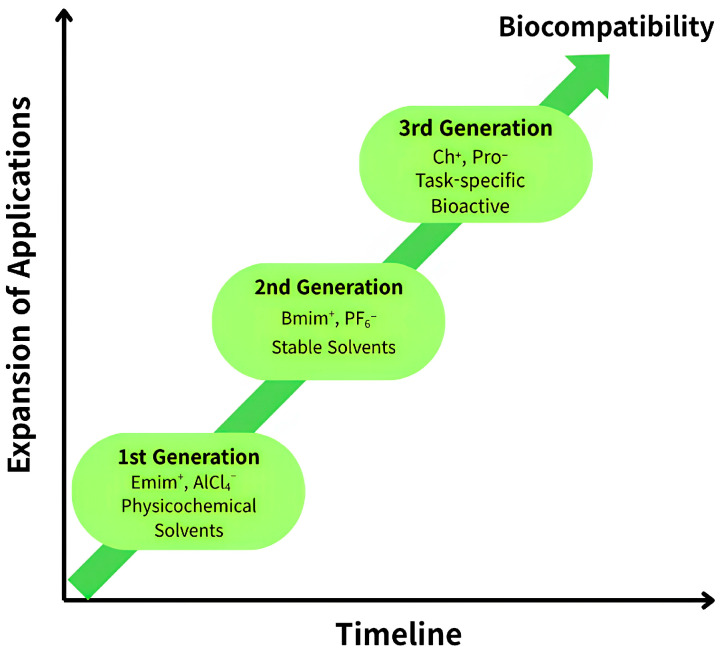
Overview, Growth Trends, and Generational Evolution in Ionic Liquid (IL) Research.

ILs have been increasingly explored as customizable drug delivery platforms owing to their ability to enhance drug solubility, permeability, and stability through rational ion design [13]. A defining feature of ILs is the precise tunability of physicochemical properties via systematic combinations of cations (e.g., imidazolium, choline) and anions (e.g., halides, acetate), which govern key parameters such as viscosity, hydrophobicity, solubility, and toxicity [10,13,14]. From a formulation perspective, the development of ILs in pharmaceutical research is commonly discussed in terms of three conceptual generations, reflecting evolutionary shifts in design priorities rather than a strictly chronological classification. The first generation primarily emphasized the use of ILs as alternative solvents, whereas the second generation focused on incorporating task-specific functionality. More recently, third-generation ILs have increasingly prioritized biocompatibility, pharmacological relevance, and environmental sustainability through rational ion selection. This section outlines the key characteristics, representative structures, and functional trends associated with commonly described IL generations, providing a structured framework for discussing their pharmaceutical applications (Figure 2). To facilitate comparison, the conceptual distinctions among IL generations—defined by shifts in formulation-driven design priorities—are summarized in Table 1. In this review, the term “generations” is used as a flexible organizational descriptor, recognizing that boundaries between generations are not standardized and that overlaps between generations are common.

The structural and functional evolution of ILs across generations, from Generation 1 (physicochemical solvents) to Generation 3 (biocompatibility/specific functions), illustrates the key ions and characteristics of each generation.

Summary of the conceptual generational classification of ILs according to dominant design priorities in pharmaceutical research. The three generations reflect shifts from physicochemical solvent-oriented ILs (1st generation) to task-specific and functionally tuned systems (2nd generation), and more recently to biocompatibility- and application-oriented ILs designed for drug delivery relevance (3rd generation). Representative cations and anions, along with key advantages and limitations, are shown to highlight how structural design strategies have progressively evolved. This classification serves as an analytical framework rather than a strict chronological or exhaustive categorization of all IL systems.

### 2.1. First-Generation ILs: Physicochemical Focused Solvents

First-generation (1st-gen) ILs trace their origins to Paul Walden’s 1914 report on ethylammonium nitrate, while the synthesis of dialkylimidazolium-based ILs in the 1980s marked a major developmental milestone. Early studies demonstrated that key physicochemical properties could be systematically tuned by modifying the cation structure, typically in combination with chloraluminate or metal halide anions. Compounds such as [C4MIM][BF4] and [C4MIM][PF6] were consequently investigated as alternatives to conventional organic solvents and were initially regarded as “green” solvents due to their high thermal stability and negligible vapor pressure. Research during this period primarily focused on physicochemical parameters, including density, viscosity, solubility, thermal stability, and vapor pressure [26,27,28,29,30,31]. Limited biological observations were also reported; for example, ethylammonium nitrate was explored as a protein crystallization additive, and selected ammonium-based ILs were shown to influence protein stability through changes in pH and solution density [32]. Certain ILs were additionally reported to modulate lysozyme amyloid formation and affect the properties of mature amyloid fibrils [33].

However, the broader application of 1st-gen ILs in biological and pharmaceutical contexts was constrained by anion-dependent hydrolytic instability, poor biodegradability, inherent aquatic toxicity, and high production costs [28,31]. Consequently, although 1st-gen ILs demonstrated promise as solvent substitutes, these limitations motivated a shift toward the development of more functionally tailored and environmentally considerate IL designs.

### 2.2. Second-Generation ILs: Biocompatibility-Oriented and Task-Specific

Second-generation (2nd-gen) ILs emerged in the 1990s as efforts expanded beyond purely physicochemical optimization to address some of the biological and environmental limitations associated with first-generation systems. Design criteria increasingly incorporated improved moisture and air stability, reduced toxicity relative to early ILs, and enhanced functional control. In this context, combinations of cations (e.g., imidazolium and quaternary ammonium) with anions such as PF_6_^−^, BF_4_^−^, and TFSI^−^ were explored primarily to minimize moisture sensitivity and improve chemical stability [28,31,34]. A defining feature of 2nd-gen ILs was the capacity to tailor properties—including thermal stability, hydrophobicity, and biological activity—through rational ion selection, enabling their use as functional materials such as biocatalytic media and metal-ion complexing agents [1,2,30,32,35]. This design flexibility led to the concept of task-specific ionic liquids (TSILs), which have been widely investigated in pharmaceutical and biomedical contexts, including DDS-related applications [24,29,32]. For example, [C_4_C_1_Im][PF_6_] was reported to enable efficient synthesis of the NSAID pravadoline, achieving yields of 90–94% [36]. Beyond synthetic utility, selected 2nd-gen ILs have also been examined for biological functionality relevant to therapeutic applications. Quaternary ammonium-based ILs were shown to exert multistep antibacterial effects via membrane disruption, suggesting potential utility in antimicrobial strategies [37]. In anticancer studies, imidazolium-based ILs such as [MIM]Cl and [Bmim][Cl] demonstrated selective cytotoxicity toward brain cancer cells (T98G) relative to healthy cells (HEK), while [Emim][Ac]-containing hydrogels promoted fibroblast proliferation (3T3-L1) while suppressing HepG2 cancer cell growth [11].

Despite these advances, the broader application of 2nd-gen ILs in DDS remains constrained by limitations including poor water solubility, which restricts aqueous formulations, high manufacturing costs associated with raw materials and purification, and ongoing concerns regarding bioaccumulation or systemic toxicity in the absence of specifically designed biocompatible functional groups [3,30,31]. Collectively, these challenges underscore the need for further refinement of IL design strategies, thereby motivating the development of third-generation systems.

### 2.3. Third-Generation ILs: Biologically Active and Environmentally Sustainable

Third-generation (3rd-gen) ILs represent a design shift toward biologically functional and application-oriented systems, aiming to address limitations encountered in earlier generations of IL-based DDS. A defining characteristic of this generation is the incorporation of ions derived from naturally occurring or biocompatible building blocks, such as choline and amino acids, which have been designed to improve biodegradability and reduce toxicity in selected formulations [31]. Notably, in many 3rd-gen ILs, the constituent ions themselves contribute biological functionality. Cations may exhibit antimicrobial activity or modulate membrane permeability, while anions can function as active API, including antibiotics or nonsteroidal anti-inflammatory drugs, or form ionic complexes with these agents [38]. Through such ion design, 3rd-gen ILs enable enhanced solubility, formulation flexibility, and route adaptability, and are often more hydrophilic than earlier generations, facilitating their exploration in oral, transdermal delivery systems [1,2,31]. 3rd-gen ILs have been widely explored as systems that combine biological functionality with tunable physicochemical attributes, and selected formulations have been reported to offer practical advantages such as simplified preparation and improved environmental acceptability [28]. In this context, 3rd-gen ILs can be designed to incorporate API-derived ions (e.g., analgesics and anti-inflammatory agents) or to enhance formulation performance, including permeability, stability, and sustained release, across multiple delivery routes such as oral, transdermal, and mucosal administration [1,27,30,37]. Representative examples include choline-based ILs, such as [Cho][Phe] and [Cho][Glu], which have been reported to enhance drug solubility while maintaining low cytotoxicity in vitro [2]. In transdermal applications, a choline-based IL–chitosan insulin patch achieved approximately a sevenfold increase in permeability and enabled sustained glycemic control, while in oral delivery, 3rd-gen IL formulations significantly improved the absorption and bioavailability of the poorly soluble anticancer drug sorafenib [39].

Despite these promising results, the performance and safety of 3rd-gen ILs remain highly structure- and formulation-dependent, and their translational potential requires systematic evaluation of long-term toxicity, regulatory classification, and manufacturing scalability. Nevertheless, by integrating biological activity with tunable physicochemical properties, 3rd-gen ILs provide a versatile formulation basis that underpins current advances in IL-enabled DDS. Building on these generational developments, the following sections examine how such systems are being implemented across diverse drug delivery routes.

## 3. Ionic Liquids-Based Drug Delivery System

The examples discussed in this review were selected to illustrate formulation-driven advantages of IL-enabled DDS, such as improvements in solubility, permeability, stability, or controlled release, based on clear structure–property–function relationships and translational relevance, rather than to provide an exhaustive survey. Emphasis was placed on platforms demonstrating clear structure–property–function relationships and higher translational relevance. These selection criteria reflect the fundamental requirements of effective DDS, which must maximize therapeutic efficacy, enhance patient compliance, and overcome physiological barriers such as the skin and cellular membranes. Many API fail to achieve therapeutic benefit due to poor bioavailability and restricted permeability, challenges that are further compounded by low API solubility and regulatory constraints on conventional organic solvents [40,41,42,43]. Since the 1980s, DDS based on micelles, liposomes, and nanoparticles has been progressively developed to improve efficacy and bioavailability [7,44]. However, these conventional approaches have intrinsic limits, such as potential toxicity, poor biodegradability, and environmental concerns. Their manufacture frequently requires large volumes of organic solvents, which increase the risks of human toxicity and environmental pollution [36,45]. Consequently, achieving sustained in vivo circulation, tissue specificity, and homogeneous drug distribution remains challenging [2,21]. Since their first application in DDS in 2008, specific classes of ILs, particularly choline-based and amino acid-derived systems, have attracted research interest due to their favorable biocompatibility profiles and their ability to enhance drug solubility and formulation stability in a structure- and formulation-dependent manner [2,46]. Specific classes of ionic liquids, including choline-based, imidazolium-based, and API-derived systems, offer formulation-specific advantages over conventional carriers, such as enhanced permeability, API–IL complex formation, precise structural tunability, and controlled biomembrane interactions. Accordingly, their applications have expanded beyond traditional transdermal and oral delivery to encompass protein delivery, nanocarrier-based systems, and microtechnology-assisted platforms, suggesting potential utility in the next-generation DDS [30,47,48,49]. In this section, IL-based drug delivery systems are discussed primarily according to delivery platform and formulation strategy rather than administration route. This organization reflects shared design principles, formulation challenges, and translational considerations that extend across different routes of administration. Where relevant, specific routes (e.g., oral, transdermal, injectable, or mucosal) are therefore treated as contextual variables within each platform-based subsection rather than as the primary basis for classification. Importantly, IL-based systems should not be regarded as universally superior to conventional DDS. Rather, their advantages are highly context-dependent, and in many scenarios, established polymeric or lipid-based carriers may remain preferable owing to their regulatory maturity, well-characterized safety profiles, and proven manufacturing scalability.

### 3.1. IL-Enabled DDS Platforms

Biological medicines (biologics), such as proteins, nucleic acids, and vaccines, represent a different therapeutic approach compared to conventional small-molecule drugs. Since the 1980s, these agents have become essential for tackling conditions like cancer and autoimmune diseases. However, biologics suffer from inherent structural instability due to their complex 3D structures, high hydrophilicity, and vulnerability to enzymatic degradation, which complicate transport, storage, and in-body delivery [50,51,52]. Specific classes of ILs, particularly those based on biocompatible ions such as choline, are regarded as promising delivery vehicles due to their tunable cytotoxicity, superior stability, enhanced permeability, and biocompatibility. It should be noted that these properties are not universal to all ILs but are highly dependent on specific cation–anion combinations, concentration ranges, and formulation context. These favorable attributes make them an attractive option for the delivery of various biomolecules, including proteins and nucleic acids. Interestingly, ILs can also be processed into alternative forms, such as films or micro/nanoparticles, for DDS applications [36,53]. Recently, innovative formulations, such as IL-based micro/nanoparticle systems and direct API-IL complexes, have been developed. These advance the field by enhancing biologic structural stability and enabling tissue-specific delivery [54].

#### 3.1.1. Nanotechnology-Enabled Delivery Systems

This subsection focuses on nanocarrier-based IL-enabled delivery platforms, with administration route considered a secondary design variable. IL-integrated nanocarrier systems have matured into versatile platforms that enhance delivery precision, prolong circulation stability, and improve in vivo performance. These systems are specifically engineered to overcome physiological barriers through coordinated control of material composition, particle size, and surface characteristics during design, synthesis, and characterization [55,56]. Nanotechnology was actively deployed in recent cancer treatment clinical trials and demonstrated significant therapeutic potential by facilitating preferential drug delivery to tumor tissues by enhancing permeability and retention (EPR) [55,56,57,58]. The integration of ILs within nanotechnology-based frameworks unlocks innovative potential for pharmaceutical and biotechnology products by facilitating precise control over the physicochemical properties of nanoscale formulations, such as surface charge, solubility, and release rate [59]. Notably, poly(ionic liquid)-based nanoplatforms and related IL–nanocarrier designs can provide formulation-specific advantages, including improved stability and tunable intermolecular interactions, which in selected systems enable controlled drug release and enhanced delivery performance [60].

##### IL-Enabled Nanocarrier and Nanoparticles for Drug Delivery

Nanocarriers and nanoparticles are central components of precision drug delivery systems, as they enhance therapeutic efficacy while minimizing off-target effects. Although these platforms are often discussed as separate categories, they share common design principles in the context of ionic liquid (IL)-based drug delivery. In both systems, ILs are incorporated to improve the solubility and stability of poorly water-soluble drugs, enable selective tissue targeting, and regulate drug loading and release behavior. Accordingly, IL-assisted nanocarriers and nanoparticles are discussed here under a unified nano-structured DDS framework.

Nano-structured delivery systems generally operate within the size range of tens to a few hundred nanometers and can facilitate targeted delivery through surface functionalization, ligand conjugation, or electrostatic modulation, which may reduce non-specific exposure to healthy tissues [61]. These systems can enhance therapeutic efficiency by protecting drugs from premature loss, improving apparent/systemic solubility, and facilitating controlled drug release at the target site [62,63]. However, many currently widely used nano-DDS platforms still face practical challenges, including high material costs, potential cytotoxicity concerns, limited drug loading capacity, and regulatory constraints driven by a preference for well-established excipients (e.g., PEG, PLGA, and HSA) in approved formulations [41,55,64,65].

In this context, recent perspectives have highlighted the emerging integration of ionic liquids with advanced nanocarrier platforms as a strategy to expand the formulation design space and functional tunability of nano DDS [66]. To address these limitations, ILs have been increasingly explored as functional components owing to their tunable chemical structures. Depending on the specific cation–anion combinations, Imidazolium-based ionic liquids, depending on their cation–anion combinations, have been shown to function as solubilizers, stabilizers, or surface modifiers in nano-DDS, enabling controlled modulation of membrane permeability and release kinetics [5,59,67]. By integrating rationally designed ILs into nanoformulations, recent studies have reported significantly enhanced entrapment efficiencies, improved physicochemical stability, and stimuli-responsive release profiles—features that are often challenging to achieve using conventional materials alone [68,69].

A representative example of an IL-assisted nano-structured DDS is the ImIL-PEG@MCM-41 system. This formulation, incorporating an imidazolium-based IL and PEG, exhibited a uniform particle size of ~150 nm and a high entrapment efficiency (EE) of 91% for lapatinib. Notably, this system demonstrated accelerated pH-dependent drug release under acidic conditions, suggesting its suitability for the tumor microenvironment (Table 2) [70].

Similarly, an alginate–clay nanocomposite incorporating an imidazolium-based IL was designed for the co-delivery of methotrexate (MTX) and ciprofloxacin (CIP). This system achieved exceptionally high EE (99% for MTX and 98% for CIP) and demonstrated pH-responsive release (Table 2) [58]. These results illustrate that imidazolium-based ionic liquids extend beyond their conventional role as solubility enhancers and function as integral structural components that impart multifunctionality to nano-DDS platforms.

Overall, while IL-enabled nano-structured systems are promising as next-generation delivery platforms, their clinical translation requires careful consideration of the specific IL type and its associated long-term biocompatibility.

##### Limitations of Nanotechnology-Based DDS

Despite the promising performance of IL-assisted nano-structured DDS, several challenges remain for clinical translation. Most notably, the safety and biocompatibility of ILs are highly structure-dependent, requiring careful ion selection and concentration control [72,73]. The lack of standardized toxicity evaluation protocols across studies complicates direct comparison and systematic risk assessment [74].

From a formulation perspective, scalability and batch-to-batch reproducibility remain significant hurdles, as nano-DDS performance is sensitive to processing conditions and additional formulation components and interfacial interactions may introduce further variability [75,76]. Moreover, the clinical translation of IL-based formulations remains limited by regulatory uncertainty. Accordingly, standardized toxicological assessment and pharmacokinetic/biodistribution evaluation are required for regulatory acceptance [77,78].

Importantly, structure–activity relationships governing IL–membrane interactions, which are also relevant to antimicrobial mechanisms, are discussed in detail in Section 4. Addressing these challenges through standardized safety criteria, rational IL design, and early regulatory alignment will be essential for advancing IL-enabled nano-DDS toward clinical application. To enable meaningful comparison and translational assessment of IL-based DDS, future studies should adopt standardized evaluation parameters, including cytotoxicity and hemocompatibility endpoints, permeability–toxicity trade-offs, pharmacokinetic relevance, formulation stability, and regulatory classification. From a translational perspective, these limitations collectively highlight persistent regulatory uncertainty, limited clinical progression, and unresolved scalability challenges that currently hinder the advancement of IL-based DDS beyond preclinical stages.

#### 3.1.2. Microtechnology-Assisted Delivery

This subsection discusses IL-integrated microtechnology-based delivery systems, organized by device architecture and formulation strategy rather than by route of administration. In this context, microtechnology-assisted platforms incorporating ILs have gained attention for their potential to support personalized therapeutic regimens. These systems address key limitations of conventional administration routes by enabling controlled drug release and minimally invasive delivery, thereby improving dosing precision and patient compliance [79,80]. Among these, reservoir-based and matrix-based systems have emerged as promising strategies for targeted delivery to anatomically challenging sites [80].

Recently, increasing attention has been directed toward integrating ILs with microtechnology platforms, such as microspheres, microneedles, and microemulsions [15,81,82]. In particular, microspheres fabricated from poly(ionic liquids) (PILs) have attracted interest as versatile carriers. Their high specific surface area and tunable polymer design flexibility support diverse applications, ranging from ion exchange to precision drug delivery [15]. The incorporation of ILs into these micro-scale systems provides unique advantages, including intrinsic antimicrobial activity and enhanced mechanical properties, which are further explored in the following subsections.

##### Ionic Liquid-Integrated Microtechnology-Based Drug Delivery Systems

Microtechnology-assisted drug delivery systems have emerged as versatile platforms for localized, minimally invasive, and sustained drug administration, addressing the limitations of conventional oral and injectable routes [79,80]. Recent research has increasingly explored integrating ILs with these systems, such as microneedles and microemulsions, to enhance functional versatility [15,81,82,83].

In microneedle-based systems, PILs offer tunable mechanical strength and intrinsic antimicrobial properties. This enables precise transdermal delivery while mitigating the infection risks associated with microchannel formation in the skin [81,83,84,85]. For example, PIL-based microneedles loaded with salicylic acid via ion exchange demonstrated synergistic anti-inflammatory and antibacterial effects, significantly suppressing *Cutibacterium acnes* growth in preclinical models (Table 3) [83].

In parallel, IL-based microemulsions provide a flexible strategy for poorly soluble drugs by exploiting the amphiphilic solvency and tunable polarity of ILs. In such systems, certain ionic liquids can function as surface-active components that govern interfacial organization and microstructural dynamics within the dispersed phases [86]. As a result, IL-based microemulsions can complement or partially substitute conventional surfactants, enhancing drug solubilization and release control [82,87,88,89,90,91]. A representative IL/o microemulsion for acyclovir delivery exhibited markedly improved skin penetration and high stability with low cytotoxicity, underscoring the potential of IL-assisted microemulsions for adaptable transdermal delivery (Table 3) [89]. Taken together, these studies suggest that IL-integrated microtechnology combines precise release control with multifunctionality, supporting its development as a next-generation therapeutic approach.

**Table 3:** Representative Ionic Liquid-Enabled Microtechnology-Assisted Drug Delivery Systems.

IL DDS Type	IL Function	Example System	Representative Outcome	Key Limitations
PIL-based microneedles	Antimicrobial matrix; ion-exchange drug loading	Imidazolium-based PIL microneedles loaded with salicylate via anion exchange [83]	Effective antibacterial and anti-inflammatory activity against *Cutibacterium acnes* (in vitro/in vivo)	Disease-specific model; limited long-term stability and scale-up data
NO-releasing PIL microneedles	Contact-active antimicrobial; sustained NO delivery	Imidazolium PIL microneedles loaded with nitric oxide [84]	Significant antifungal and antibiofilm activity; accelerated wound healing in vivo	NO stability and long-term biosafety remain unclear
IL/o microemulsion	Drug solubilization; dermal permeation enhancement	Imidazolium-based IL incorporated into IPM microemulsion for acyclovir delivery [89]	Nano-sized droplets (~20 nm); enhanced skin deposition and transdermal flux	Imidazolium-related toxicity; limited biodegradability and long-term safety
IL/o microemulsion	IL-based reservoir for dermal delivery	BMIMBr-based IL/o microemulsion for 5-fluorouracil [92]	Up to 4-fold increase in dermal penetration; improved therapeutic efficacy	Potential toxicity and regulatory concerns of imidazolium ILs

This table summarizes the application of ILs in micro-scale platforms, such as microneedles and microemulsions, focusing on their roles in antimicrobial activity and skin permeation enhancement, along with associated manufacturing and safety challenges. Abbreviations: PIL, Poly(ionic liquid).

##### Limitations of Microtechnology-Based DDS

Despite the functional advantages of IL-assisted microtechnology, several hurdles related to safety, scalability, and regulatory acceptance must be addressed to enable clinical translation [77,93,94].

For microneedle-based systems, ensuring sufficient mechanical robustness and batch-to-batch reproducibility remains a major barrier, especially under large-scale manufacturing conditions [93,94,95,96]. Variations in material selection and PIL composition can significantly influence insertion behavior and mechanical strength, directly impacting drug loading capacity and release kinetics [93,94,96].

In IL-based microemulsion systems, formulation complexity and extreme sensitivity to component ratios present significant challenges. Such sensitivity to compositional parameters can compromise structural stability and reproducibility during scale-up, consistent with previous reports on complex emulsion systems [97].

Furthermore, while ILs can reduce the need for conventional surfactants, comprehensive long-term safety evaluations remain mandatory. Since many ILs are not yet established as standard excipients, achieving regulatory alignment for IL-containing formulations is a critical step for future clinical use [1,77]. Addressing these challenges through rational design and standardized evaluation criteria will be essential for advancing IL-integrated microtechnology toward practical applications [77,95].

#### 3.1.3. Biomacromolecule-Based Delivery

This subsection addresses IL-enabled delivery strategies for biomacromolecules, with emphasis on formulation and stabilization challenges rather than route-specific classification. Biomacromolecular therapeutics, including proteins, nucleic acids, peptides, and vaccines, present unique formulation challenges due to their structural complexity and susceptibility to degradation, and IL-based approaches have been explored to improve their stability, solubility, and delivery performance [52,98]. These macromolecules possess complex three-dimensional structures and high hydrophilicity. Due to their susceptibility to enzymatic degradation and structural inactivation in vivo, the development of appropriate DDS is essential to preserve their functional integrity and therapeutic performance [51].

In this context, ILs are increasingly explored as alternative delivery vehicles. Compared to traditional solid formulations, API-derived ionic liquid systems and choline-based ionic liquids have been reported to provide improved solubility control, enhanced biomembrane permeability, and increased physicochemical stability, depending on the specific ion pair and formulation context [18,41]. Crucially, IL toxicity and biocompatibility are highly dependent on cation–anion composition and formulation context, rather than being universal across all IL classes. Moreover, the tunability and formulation versatility of ILs support their investigation as candidate materials for next-generation biopharmaceutical formulations [52,72]. Consequently, ILs can fulfill diverse roles, including preserving protein and nucleic acid structures, enhancing resistance to enzymatic degradation, and improving intracellular delivery, either as standalone systems or in combination with nano- and microtechnology-based carriers. In addition, ILs have been investigated as stabilizing agents and immunomodulatory components in vaccine formulations, highlighting their emerging potential as a versatile approach for a range of biomacromolecule-based drug delivery strategies.

##### Protein-Based Drug Delivery

Therapeutic proteins have been a core biopharmaceutical segment since the 1980s but are fundamentally limited by poor oral bioavailability (typically ≤ 2%). This limitation stems from their sensitivity to gastrointestinal (GI) enzymes, extreme pH levels, and inherent structural instability [47,99].

In this context, ILs have been investigated as stabilizing agents capable of inhibiting protein denaturation. Choline-based and fluorinated ionic liquids have been shown to prevent protein unfolding and preserve native folded structures and biological activity by modulating protein–solvent interactions [13,32,100]. These systems can improve safety and facilitate long-term storage; studies have demonstrated that certain IL formulations maintain protein integrity for up to two months at ambient temperature and at least four months when refrigerated [32,99]. Furthermore, ILs can be leveraged as vaccine stabilizers to suppress degradation under adverse environmental conditions [100].

A representative study fabricated multifunctional poly(lactic-co-glycolic acid) (PLGA) nanoparticles using deoxycholic acid (DCA) and a choline-based IL to encapsulate human growth hormone (HGH). This system achieved high encapsulation efficiency, stable release, and excellent biocompatibility. Notably, it demonstrated a 2.1-fold improvement in oral bioavailability compared to controls, alongside a storage stability of two months at room temperature (Table 4) [99]. However, protein stabilization in IL-containing systems is highly dependent on the specific cation–anion combinations, IL concentration, and exposure conditions, and certain ILs may induce protein denaturation or aggregation under unfavorable conditions.

In summary, rationally designed ILs, when appropriately selected and formulated, have been proposed as a precision formulation approach capable of improving structural stability and biological activity, thereby broadening the administration routes for protein therapeutics.

##### Nucleic Acid-Based Drug Delivery

Nucleic acids, such as DNA and RNA, regulate gene expression and represent a central modality for treating complex diseases. Specifically, siRNA, miRNA, and plasmid DNA offer broad therapeutic potential, including gene modulation, gene therapy, and molecular vaccine development [53,106]. However, the clinical success of these therapies depends on meeting stringent requirements for purity, structural stability, and efficient intracellular delivery [20,107,108]. DNA, in particular, requires a stabilizing medium capable of preserving its complex conformation during long-term storage [109].

In this context, ILs have been explored as delivery vehicles and stabilizing media for nucleic acid-based therapeutics. ILs can stabilize the nucleic acid–solvent interface through a combination of hydrogen bonding, electrostatic interactions, and hydrophobic effects. These interactions have been reported to enhance resistance to enzymatic degradation and improve cell membrane permeability in aqueous environments [107,108,109]. Choline-based ILs have received particular attention due to their relatively low toxicity and favorable biocompatibility profiles. Specific choline-based IL formulations have been reported to enhance intracellular nucleic acid delivery and improve transfection efficiency [1,53]. Furthermore, IL-based formulations have demonstrated utility as delivery vehicles in selected nucleic acid delivery contexts [110].

A representative study (Table 4) showed that incorporating citric acid into choline-based ILs improved DNA structural stability by increasing the zeta potential of the [Ch]IL–DNA complex. This optimized formulation was associated with enhanced delivery efficiency, attributed to improved solubility and increased intracellular uptake while maintaining good biocompatibility [104]. Collectively, these findings suggest that specific IL-based systems can function as effective in vivo delivery platforms, extending their role beyond simple preservation media and supporting the further development of gene therapies and RNA-based vaccines.

##### Vaccine-Based Drug Delivery

In vaccine or adjuvant delivery platforms, antigens can be efficiently presented to APCs, thereby eliciting both humoral and cellular immune responses [111]. Current vaccine formulations are typically developed in various formats, such as suspensions, nanoparticles, or emulsions, and are administered via multiple routes, including oral, intramuscular, and transdermal delivery [1,100]. Maintaining the structural integrity of biomacromolecular antigens is essential, as they are susceptible to degradation or aggregation induced by changes in moisture, temperature, and pH [52].

In particular, vaccines based on split or purified antigens often exhibit limited intrinsic immunogenicity, necessitating the use of adjuvants and delivery platforms to enhance immune activation and antigen availability; however, even with adjuvantation, the induction of robust type-1 cellular immune responses, including Th1-skewed and CD8^+^ T-cell immunity, remains challenging, while the inherent physical instability of vaccine antigens further necessitates cold-chain storage and transportation, increasing formulation, distribution, and logistical burdens [52,111,112]. In this context, IL-based systems have been explored to enhance vaccine stability and immunogenicity by stabilizing antigens, improving cell membrane permeability, and facilitating antigen penetration, owing to their tunable molecular structures and favorable biocompatibility profiles [113]. Such formulations have been investigated as penetration enhancers and stabilizing matrices to mitigate antigen degradation [43,100].

A representative example is the use of choline-based ionic liquids, such as [Cho][Cl] and [Cho][SO_4_], which markedly improved the stability of inactivated viral antigens while maintaining immunogenicity, highlighting their potential as vaccine-stabilizing excipients (Table 4) [102]. This formulation achieved balanced Th1/Th2 immune responses, illustrating the potential of IL-inspired formulation strategies to modulate immune responses.

Overall, these findings indicate that IL-based delivery concepts may help address key limitations of conventional vaccine formulations by improving antigen stability and immunogenicity, thereby supporting the development of heat-stable and advanced vaccine platforms.

##### Limitation of Biomacromolecule-Based Delivery

Despite the increasing interest in ionic liquid (IL)-enabled biomacromolecule-based drug delivery systems, their clinical translation remains limited by biomolecule-specific variability and unresolved safety and regulatory concerns inherent to IL-based formulations.

For protein-based delivery, ILs can stabilize native conformations by modulating protein–solvent interactions; however, these effects are highly dependent on protein structure, ion-pair chemistry, IL concentration, and hydration state, and non-optimized IL environments may instead induce unfolding, aggregation, or loss of biological activity. In addition, IL-induced changes in viscosity and osmolality can compromise injectability, particularly for parenteral protein formulations [50,109].

For nucleic acid-based delivery, ILs have been shown to stabilize DNA and RNA and enhance resistance to enzymatic degradation; nevertheless, physicochemical stabilization does not guarantee predictable in vivo performance, as serum interactions, cellular uptake, intracellular trafficking, and clearance remain insufficiently controlled by IL formulations alone [107].

In vaccine-based delivery, IL-based and IL-inspired systems can improve antigen stability and modulate immune responses; however, uncertainties persist regarding immune mechanisms, dose-dependent reactogenicity, long-term safety, and regulatory classification, particularly for hybrid systems [113]. Overall, effective translation of IL-based biomacromolecule DDS requires biomolecule-specific optimization, comprehensive safety evaluation, and clearer regulatory alignment beyond proof-of-concept studies.

### 3.2. Route-Specific Drug Delivery Using Ionic Liquid

For clarity, mucosal delivery routes (e.g., buccal, nasal, and sublingual) are discussed separately from conventional oral delivery, as they differ in absorption mechanisms, physiological barriers, and regulatory considerations. This distinction is particularly relevant for DDS design, which aims to maximize therapeutic efficacy while minimizing systemic side effects. However, conventional DDS often suffer from non-uniform drug distribution, low bioavailability, and limited targeting efficiency, which can result in unintended systemic exposure [114]. Addressing these challenges requires a route-specific design strategy that accounts for drug physicochemical properties, biological barriers, administration routes, and regulatory considerations [115]. ILs, owing to their structurally tunable cation–anion combinations, have attracted attention as adaptable components in pharmaceutical formulations [10]. Depending on their chemical composition, ILs can contribute to functions such as enhanced membrane permeation, solubilization of poorly soluble drugs, stabilization of labile therapeutics, modulation of toxicity, and control of release kinetics. Importantly, these functions are route-dependent and must be evaluated within the context of specific delivery pathways, including transdermal, oral, and injectable administration. Accordingly, ILs should be regarded not merely as alternative solvents, but as functional formulation components that can be selectively engineered to address route-specific limitations of existing DDS [10,116].

#### 3.2.1. Transdermal Drug Delivery

Transdermal drug delivery (TDD) represents an important non-invasive administration route in pharmaceutical drug delivery. By exploiting the large surface area of the skin (approximately 1.5–2.0 m^2^), TDD can bypass hepatic first-pass metabolism, thereby improving drug bioavailability and avoiding degradation under gastrointestinal conditions. In addition, its suitability for painless self-administration significantly enhances patient compliance, particularly in the management of chronic diseases [2,32,61,117,118].

Despite these advantages, the skin constitutes a highly effective multilayered physiological barrier against exogenous substances. The outermost stratum corneum (SC), composed of corneocytes embedded in a densely packed lipid matrix, serves as the primary physicochemical barrier and severely restricts drug permeation [39,119,120]. Hydrophilic compounds are hindered by lipid-rich intercellular domains, whereas hydrophobic molecules encounter resistance from tightly packed keratinized structures. Conventional chemical penetration enhancers (CPEs), such as ethanol and sulfoxides, have been employed to disrupt SC lipid organization; however, their clinical applicability is limited by skin irritation and safety concerns [43]. Moreover, the heterogeneous nature of the skin, characterized by the coexistence of hydrophilic and hydrophobic regions, necessitates delivery systems capable of accommodating both environments [20]. In this context, ILs, composed of organic cations and anions with tunable polarity and intermolecular interactions, can interact with SC lipids and proteins, resulting in transient modulation of SC barrier properties [120]. Several studies have reported that specific IL formulations increase SC lipid fluidity and promote intercellular diffusion pathways, supporting their potential as penetration-enhancing components compared with conventional CPEs [6,121]. Notably, choline-based ionic liquids have been shown to significantly enhance the transdermal delivery of insulin while maintaining skin compatibility [122]. Related IL-mediated nanovesicular systems further demonstrate the feasibility of IL-containing vesicular platforms for macromolecular transdermal delivery (Table 5) [123].

Overall, IL-based strategies represent a promising yet structure-dependent approach for transdermal drug delivery. Future advances in this field will rely on systematic structure–activity relationship (SAR) studies and the integration of ILs into composite delivery systems that balance permeability enhancement with long-term skin safety.

#### 3.2.2. Oral Drug Delivery

Oral drug delivery is widely favored due to its simplicity, non-invasiveness, favorable safety profile, affordability, and ease of self-administration, and remains the most commonly used route in clinical practice [32,43]. Despite these advantages, oral administration is widely regarded as one of the most formulation-challenging routes, particularly for modern drug candidates with poor aqueous solubility and limited permeability, which often result in low and variable bioavailability [42,124].

In this context, ILs have emerged as formulation-enabling materials for oral drug delivery, as their ion-level tunability allows modulation of solubility, stability, and interfacial interactions beyond conventional excipients [36,77]. Importantly, oral delivery involves distinct biological interfaces, most notably oral mucosal delivery and gastrointestinal (GI) tract-mediated absorption, which differ in their dominant barriers and pharmacokinetic endpoints. Accordingly, this section discusses IL-enabled strategies for oral mucosal and GI drug delivery separately, with emphasis on route-specific mechanisms and limitations.

##### Oral Mucosa Drug Delivery

Oral mucosal delivery is a non-invasive route that facilitates drug absorption through the highly vascularized oral mucosa. This pathway enables rapid systemic uptake while partially avoiding gastrointestinal degradation and hepatic first-pass metabolism. Although suitable for both local and systemic therapies, its clinical application is challenged by salivary washout, a limited absorptive surface area, and enzymatic activity, which collectively reduce residence time and hinder efficient epithelial transport [125,126].

ILs have emerged as versatile materials for oral mucosal delivery, offering tunable ion-pair chemistry and controllable interfacial interactions. Through rational cation–anion selection, ILs can modulate API crystallinity and apparent solubility. Furthermore, API–IL strategies provide formulation flexibility even under conditions of short residence time. However, as IL–membrane interactions are highly structure-dependent, IL-enabled oral mucosal DDS should be treated as route-specific systems that require the simultaneous optimization of ion design and local biocompatibility [3,127].

##### Gastrointestinal (GI) Drug Delivery

GI-mediated absorption represents the dominant pathway of oral drug delivery, benefiting from the large surface area and rich vascularization of the small intestine, which support efficient systemic and localized uptake [128,129].

However, GI delivery is challenged by a dynamic luminal environment—including pH variations, enzymatic/bile activity, and mucus barriers—alongside variable transit times, which can destabilize drugs and lead to inconsistent absorption [130,131]. Furthermore, epithelial barriers and transporter-mediated efflux (e.g., P-glycoprotein) can further limit intestinal uptake, particularly for poorly soluble or permeability-limited compounds [131].

In this context, ionic liquids (ILs) have emerged as promising formulation-enabling materials to overcome these limitations through rational ion-pair design [127]. By modulating API crystallinity and intermolecular interactions, API-derived ionic liquids and choline-based IL formulations can enhance apparent solubility, accelerate dissolution under GI conditions, and favorably influence drug–membrane partitioning [3,127]. Nevertheless, as IL–membrane interactions are highly structure-dependent and may impact epithelial integrity, IL-enabled GI delivery must be approached as a route-specific strategy that requires simultaneous optimization of ion composition, concentration, and intestinal biocompatibility [3].

#### 3.2.3. Injectable Drug Delivery

Injection-based drug delivery plays a central role in the treatment of acute conditions and high-risk diseases due to its rapid onset of action, precise dose control, and high bioavailability [132]. Selection of an appropriate injectable route requires careful consideration of safety, therapeutic efficacy, patient compliance, and cost-effectiveness [133]. Intravenous (IV) administration enables immediate systemic exposure by bypassing absorption barriers and first-pass metabolism, allowing tight control over plasma drug concentrations [132]. In contrast, subcutaneous (SC) administration offers a less invasive alternative that can support sustained drug release, as exemplified by insulin formulations, while also being applicable to vaccine delivery with reduced pain and procedural complexity [133,134]. Despite these advantages, injectable formulations are frequently limited by poor aqueous solubility, insufficient physicochemical stability, and adverse reactions, including local tissue irritation and undesired immunological responses [1]. Such challenges are particularly critical for parenteral administration, where formulation robustness and biocompatibility are essential for clinical translation.

In this context, ILs provide a molecular-level strategy to address formulation challenges associated with injectable delivery. As discussed above, ILs can enhance the solubility of poorly soluble drugs and improve formulation stability through strong ionic interactions and broad solvent compatibility [1,10]. Furthermore, their tunable hydrophilic–hydrophobic balance enables the rational design of injectable platforms, including polymer-based systems and nanocarriers, that support controlled and sustained drug release. Representative studies support these advantages. Choline-based IL-loaded deoxycholic acid (DCA) nanoparticles and imidazolium-based IL-functionalized mesoporous silica nanoparticle (MSN) systems have demonstrated improved delivery efficiency and acceptable biocompatibility in injectable formulations (Table 5) [35,40]. In addition to their formulation-enhancing roles, certain ILs have been reported to exhibit intrinsic antimicrobial activity, which may offer auxiliary benefits in specific injectable applications, although this property requires careful consideration with respect to safety and dose control. Overall, IL-based strategies expand the design space of injectable drug delivery systems by enabling improved solubility, stability, and control over drug release. Route-specific safety, immunogenicity, and regulatory considerations are addressed in the limitations section.

#### 3.2.4. Limitations of Drug Delivery Systems Across Different Administration Routes

Despite the formulation advantages of IL-based DDS, clinical translation remains constrained by route-dependent safety, biocompatibility, and regulatory challenges.

For transdermal delivery, ILs can enhance permeation across the stratum corneum; however, excessive permeation may disrupt skin barrier integrity and induce local irritation due to interactions with lipid and protein components of the skin. In addition, comprehensive safety profiles and in vivo tolerability data for prolonged dermal exposure to permeation-enhancing IL formulations remain limited, necessitating careful safety evaluation and exposure control strategies [78,135].

For oral administration, ILs—particularly API–IL strategies—have been explored to improve the solubility and oral exposure of poorly soluble small-molecule drugs, as demonstrated by pharmacokinetic studies such as favipiravir-based ILs [18]. However, reviews on pharmaceutical ILs emphasize that the same physicochemical features underlying enhanced dissolution and permeation also require careful consideration of gastrointestinal tolerability and structure-dependent biological effects for each ion pair [1]. Moreover, oral performance is strongly influenced by IL structure, concentration, and formulation context, underscoring the need for systematic in vivo pharmacokinetic and safety evaluation to support translational relevance [77].

For injectable delivery, parenteral administration imposes stringent safety requirements, as systemic exposure amplifies risks related to cytotoxicity, hemocompatibility, and immunogenicity. Although ILs can improve solubility and stability of poorly soluble API, structure–activity relationships governing biosafety—such as alkyl chain length-dependent toxicity—remain insufficiently characterized, and regulatory uncertainties persist regarding impurity control and acceptable safety margins [16,73].

Overall, the translational potential of IL-based DDS hinges on rational ion-pair selection, comprehensive safety profiling, and standardized regulatory frameworks that account for route-specific tolerability and performance across transdermal, oral, and injectable platforms.

## 4. Antimicrobial Properties of Ionic Liquids

Research on ILs has expanded beyond their established applications in chemistry and materials science toward exploration in the life science and pharmaceutical fields [136,137]. Owing to their low vapor pressure, high thermal stability, and structurally tunable ionic architectures, ILs have attracted attention as multifunctional materials with potential antimicrobial relevance. However, accurate toxicity assessment remains a critical prerequisite for biological application, as the same membrane-active properties responsible for antimicrobial effects may also induce undesirable cytotoxicity. Indeed, several classes of ILs exhibit toxicity toward aquatic and biological systems, highlighting that antimicrobial performance cannot be considered independently of safety. Accordingly, recent studies have increasingly focused on systematically defining Structure–Activity–Toxicity Relationships (STARs) to establish rational design criteria that balance antimicrobial efficacy with biocompatibility and environmental safety [137,138,139]. When appropriately engineered, specific cation–anion combinations enable ILs to modulate antimicrobial behavior through mechanisms such as biofilm inhibition, membrane disruption, and altered membrane permeability. Importantly, this structure-governed mode of action distinguishes ILs from conventional antibiotics that target discrete biochemical pathways and provides a framework for evaluating both their antimicrobial potential and associated biological risks [140,141]. In this context, the antimicrobial properties of ILs should be regarded as structure-dependent functionalities rather than universally desirable attributes. Accordingly, this section reviews IL antimicrobial mechanisms with emphasis on structure–activity relationships relevant to application-specific design. Table 6 summarizes representative microbial studies showing how cation structure, alkyl chain length, and cation–anion combinations govern antimicrobial behavior and Gram-type-dependent responses. In general, long-chain cholinium- and imidazolium-based ILs exhibit enhanced membrane-disruptive and antibiofilm activities, particularly against Gram-positive strains.

### 4.1. Antimicrobial Activity and Applications

Antimicrobials inhibit or eradicate pathogenic microorganisms and remain indispensable in modern medicine; however, widespread antibiotic use since the 1970s has accelerated the emergence of multidrug-resistant (MDR) strains, creating a major global health challenge [141,147]. Compounding this issue, many antibiotic candidates in current development are susceptible to existing resistance mechanisms or suffer from intrinsic pharmacological limitations that restrict therapeutic efficacy [148].

In this context, ionic liquids (ILs) and PILs have been explored as complementary antimicrobial platforms rather than direct replacements for conventional antibiotics. Unlike low-molecular-weight antimicrobials that target specific biochemical pathways, selected PIL systems exert antimicrobial effects primarily through physical mechanisms, particularly membrane disruption, which is considered less prone to rapid resistance development due to the absence of single molecular targets [148,149]. Consequently, rapid antimicrobial responses have been reported for certain PIL architectures.

In parallel, active pharmaceutical ingredient–ionic liquid (API–IL) formulations have emerged as a strategy to enhance the performance of existing antibiotics by improving solubility, permeability, and stability. For example, an ampicillin-based IL exhibited enhanced activity against resistant *Escherichia coli* and methicillin-resistant *Staphylococcus aureus* compared with its corresponding halide salts, illustrating how formulation-driven effects can modulate antimicrobial efficacy [150]. Beyond API–ILs, synergistic antimicrobial effects have also been reported when ILs are combined with conventional antibiotics; notably, long-alkyl-chain imidazolium- and pyrrolidinium-based ILs significantly enhanced the activity of colistin against Gram-negative bacteria, highlighting their potential role as adjuvant components in antimicrobial strategies [151].

Collectively, IL- and PIL-based systems may function as complementary platforms for antimicrobial delivery and adjuvant design; however, their practical applicability depends on careful control of structure-dependent membrane activity and rigorous evaluation of safety, resistance risk, and translational feasibility, rather than on antimicrobial potency alone.

### 4.2. Mechanistic Insights into Ionic Liquids-Induced Microbial Inhibition

Conventional antibiotics typically target discrete cellular processes, such as cell wall synthesis, protein biosynthesis, or essential metabolic pathways, which can facilitate resistance development upon repeated exposure. In contrast, the antibacterial activity of ILs arises from multifaceted, structure-dependent mechanisms that differ fundamentally from those of conventional antibiotics.

At the cellular level, cationic IL components interact electrostatically with negatively charged bacterial surfaces, leading to perturbation of the phospholipid bilayer and membrane-associated proteins. These membrane-centered interactions increase permeability, promote leakage of intracellular contents, and induce osmotic imbalance, ultimately resulting in growth inhibition or cell death in susceptible organisms [148,152,153]. In some cases, IL exposure has also been associated with secondary intracellular stress responses, including reactive oxygen species (ROS) accumulation, in a structure- and concentration-dependent manner [152,154]. The antimicrobial activity of ILs is strongly governed by cationic structure, particularly alkyl chain length, which modulates hydrophobicity and membrane affinity [155,156]. In general, increasing alkyl chain length beyond approximately four carbon atoms enhances membrane interactions and correlates with reduced minimum inhibitory concentration (MIC) values in multiple bacterial models [137,139]. The chemical identity of the cation further influences activity; for example, imidazolium- and pyridinium-based ILs often exhibit higher antimicrobial potency than quaternary ammonium analogues due to differences in aromaticity, charge distribution, and membrane interaction profiles [146,147,157,158]. Although anions are generally considered secondary contributors, they can significantly influence biofilm inhibition, membrane permeability, and solubility, thereby modulating overall antimicrobial performance. Appropriate selection of anions such as NTf_2_^−^, HSO_4_^−^, and SCN^−^ has been shown to alter membrane interactions and stability in specific IL systems [147,152,159,160,161]. Taken together, cation-focused structural optimization combined with rational anion pairing represents a central design strategy governing the antimicrobial activity, selectivity, and safety profiles of ILs. Importantly, these same structure–activity relationships that drive antimicrobial efficacy also define toxicity thresholds, underscoring the need for controlled, application-specific IL design.

### 4.3. Differential Effects on Gram-Positive and Gram-Negative Bacteria

ILs exhibit distinct antimicrobial activity profiles against Gram-positive (G^+^) and Gram-negative (G^−^) bacteria, reflecting selective interactions with differences in cell envelope architecture rather than surface charge alone [138,152,156]. In general, Gram-negative bacteria display higher tolerance to IL exposure due to the presence of an outer membrane barrier that restricts penetration of hydrophobic or ionizable compounds [162,163]. Specifically, Gram-negative bacteria possess a complex double-membrane architecture composed of outer and inner membranes, which limits intracellular access of ILs and attenuates antimicrobial activity [162,163]. The imidazolium-based IL [OMIM][NO_3_] exhibited potent antibacterial activity against *Staphylococcus aureus* (MIC = 0.097 g/L), whereas the pyridinium-based ionic liquid [HPY][NO_3_] showed pronounced anti-adhesive activity with comparatively weaker bactericidal effects, suggesting a predominantly surface-associated inhibitory behavior [146]. Structure-dependent trends further support the central role of membrane interactions in IL-mediated antimicrobial activity. Within the 1,3-dialkylimidazolium series ([BMIM][Br], [HMIM][Br], [OMIM][Br]), increasing alkyl chain length has been associated with enhanced membrane interaction and permeability, resulting in increased antimicrobial activity, with *B. subtilis* exhibiting particularly high sensitivity to these changes [158,164]. However, Gram-negative bacteria can activate compensatory responses to membrane-active agents; for example, adaptive increases in membrane fluidity through the synthesis of trans fatty acids have been reported to partially restore membrane stability and enhance bacterial survival under IL exposure [154].

Collectively, these observations indicate that the antimicrobial efficacy of ILs is governed by specific intermolecular interactions with bacterial cell envelope structures rather than by nonspecific chemical toxicity. Accordingly, rational molecular design strategies that account for the structural and physiological characteristics of target bacterial membranes are essential, both for understanding antimicrobial behavior and for evaluating the implications of membrane-active ILs in drug delivery system design.

### 4.4. Limitations and Implications of Antimicrobial Ionic Liquids in DDS Design

Despite the antimicrobial activity reported for selected ILs structures, several limitations constrain their practical application. Antimicrobial efficacy is strongly structure-dependent and frequently increases with alkyl-chain length and lipophilicity, but these same trends are also associated with increased cytotoxicity, indicating a fundamental activity–toxicity trade-off [158,165].

Moreover, a large fraction of antimicrobial ILs exert their effects through membrane-active mechanisms (e.g., permeabilization, pore formation, and disruption of membrane integrity), which helps explain rapid antibacterial action but simultaneously raises safety concerns because membrane disruption is not intrinsically selective for bacterial membranes [19,152,166]. Consequently, systemic antimicrobial use is often unrealistic for many IL chemistries, and practical applications are more defensible when ILs are confined to localized contexts (e.g., topical/biomaterial-associated settings) or used as formulation components where exposure can be controlled [74,152].

Bacterial envelope architecture further limits broad antimicrobial generalization. Gram-negative bacteria possess an additional outer membrane barrier that restricts penetration of many hydrophobic or ionizable agents, frequently resulting in higher tolerance relative to Gram-positive species [167,168]. In addition, microbes can display adaptive responses under IL stress, and recent reviews emphasize that such resilience mechanisms should be considered when interpreting antimicrobial outcomes and when designing IL structures for biological use [169].

Within the context of drug delivery systems, these constraints indicate that antimicrobial activity should not be treated as a primary objective of IL-based DDS. Instead, it is best framed as a secondary, structure-dependent manifestation of membrane interaction that must be carefully managed to balance permeability enhancement with safety and translational feasibility [114,165].

## 5. Challenges and Limitations of Ionic Liquid-Based Biomedical Applications

Beyond the platform-specific limitations discussed above, several cross-cutting challenges and translational barriers are consolidated below. Although ionic liquids (ILs) exhibit promising physicochemical properties for drug formulation and delivery, their toxicological and environmental profiles remain important translational barriers. The acute and chronic toxicity of ILs has been widely documented across diverse biological systems, where structural features such as cation headgroups and alkyl chain length can markedly influence cytotoxicity and biosafety outcomes, with many ILs demonstrating higher toxicity than baseline models predict and limited cellular metabolism of ILs observed in vitro, underscoring structure-dependent hazard potential [170]. Moreover, the biodegradability of many ILs is still poorly understood, with data limited in scope, and early-generation ILs often suffer from low biodegradability and potential persistence in terrestrial and aquatic environments, challenging assumptions of environmental benignity [114,171]. Reviews of IL ecotoxicity further indicate that low volatility does not necessarily translate into reduced ecological risk, and systematic investigations of environmental fate and biological effects of ILs highlight the need for context-specific assessment frameworks [172,173]. Additionally, despite interest in biocompatible IL design strategies, many ILs may remain inherently toxic or non-biodegradable, necessitating the development of safer alternatives and comprehensive hazard evaluation [1]. From a regulatory standpoint, ILs are not yet clearly classified within existing pharmaceutical frameworks, which complicates their development pipeline and emphasizes the need for early regulatory alignment, standardized toxicity assessment, and environmental impact evaluation to advance IL-based technologies toward clinically and translationally viable use.

## 6. Conclusion and Future Perspectives

ILs have emerged as multifunctional formulation components in advanced drug delivery systems due to their tunable physicochemical properties and ability to modulate biomolecular and interfacial interactions. Rather than serving as universally applicable delivery agents, their value lies in application-specific adaptability across nanocarrier-based platforms, microtechnology-assisted systems, and biomacromolecule formulations.

A central theme of this review is the structure-dependent, membrane-active behavior of ILs, which underpins both delivery enhancement and antimicrobial effects. This dual functionality highlights the need to balance efficacy and safety, as IL–membrane interactions govern therapeutic performance as well as toxicity. Accordingly, ILs should be regarded as formulation-enabling materials whose successful use depends on rational molecular design and rigorous safety evaluation.

Despite significant progress, clinical translation remains limited by unresolved challenges, including insufficient long-term toxicity data, a lack of standardized evaluation frameworks, scalability issues, and regulatory ambiguity. Future advances will depend on refining structure-guided design strategies, establishing standardized benchmarks, and aligning formulation innovation with translational and regulatory requirements.

## Figures and Tables

**Table 1:** Conceptual classification of ionic liquid generations based on evolving design priorities for pharmaceutical applications.

Generation of ILs	Representative Cations	Representative Anions	Key Advantages	Key Limitations
1st generation (physicochemical solvent-oriented)	Imidazolium (e.g., [C4mim]+), pyridinium	[AlCl4]−/chloroaluminate, PF6^−^, BF4^−^	Alternative solvent behavior with high thermal stability and negligible vapor pressure	Hydrolytic instability (anion-dependent); low biodegradability; toxicity concerns limiting pharmaceutical use [22,23]
2nd generation (task-specific and functional control)	Imidazolium, quaternary ammonium (NR4+)	BF4^−^, PF6^−^ (often also TFSI− in practice)	Tunable physicochemical properties enabling task-specific functionality and improved stability	High production cost; limited aqueous compatibility; structure-dependent toxicity [22,24]
3rd generation (biocompatibility- and application-oriented)	Choline+, amino acid-based cations (representative class)	Organic acids/amino acids, API-derived anions (API-IL concept)	Improved biocompatibility and pharmacological relevance; enhanced solubility and formulation flexibility for DDS applications	Limited long-term safety and regulatory data; performance highly formulation-dependent [1,25]

**Table 2:** Representative Ionic Liquid-Enabled Nanocarrier-Based Drug Delivery Systems.

IL DDS Type	IL Function	Example System	Representative Outcome	Key Limitations
IL-polymer nanoparticle hybrid	Solubility enhancement; sustained release	Rutin-loaded PLGA NPS with choline-based ILs ([Cho][Phe], [Cho][Glu] [69]	NP size 250–300 nm; ζ −40 mV; EE ≤ 76%; sustained release ~85% (72 h); no significant cytotoxicity (HaCaT cells)	Limited long-term and scale-up data; primarily in vitro/ex vivo studies; in vivo IL safety unverified
Zwitterionic IL-modified polymeric NPs	Surface charge modulation; reduced nonspecific interactions	ZIL-coated PEG-PLGA NPs [71]	Altered surface properties; enhanced cellular interaction; low hemolysis	Drug loading and in vivo PK/efficacy not fully assessed
IL-functionalized mesoporous silica NPs	Drug loading and pH-responsive release control	Imidazolium-IL-PEG@MCM loaded with lapatinib [70]	EE: ~91%; pH-responsive release (48 h)	Predominantly in vitro studies; manufacturing reproducibility not discussed.
Stimuli-responsive PIL polymeric nanoparticles	High drug loading; pH/light-triggered release	Amphiphilic block copolymer NPs containing PIL block (P[VHim]NTf_2_) loaded with doxorubicin (DOX) [65]	NP size ~40–80 nm; LC up to ~70%; dual-responsive release (pH- and UV-triggered); enhanced tumor cell uptake; improved antitumor efficacy (in vitro/in vivo)	Predominantly cancer-focused models; external light stimulus limits translational feasibility; long-term safety and scalability are not addressed.
IL-modified polymer–clay nanocomposite	Charge-mediated high drug loading; pH-responsive co-delivery	Imidazolium-based IL/alginate/clay nanocomposite co-loaded with methotrexate (MTX) and ciprofloxacin (CIP) [58]	Particle size ~70 nm; EE (MTX ~99%, CIP ~98%); pH-responsive release; enhanced anticancer and antibacterial activity compared with free drugs	Mainly in vitro evaluations; complex multicomponent formulation; long-term biocompatibility and manufacturing reproducibility not fully assessed

This table presents various IL-integrated nanocarriers, detailing the specific roles of ILs in enhancing drug solubility, stability, and stimuli-responsive release, while identifying current challenges for clinical translation. Abbreviations: EE, Entrapment Efficiency; LC, Loading Content; NP, Nanoparticle; PK, Pharmacokinetics; ζ, Zeta potential; [Cho][Phe], Choline phenylalanine; [Cho][Glu], Choline glutamate; DOX, Doxorubicin; MTX, Methotrexate; CIP, Ciprofloxacin; MCM, Mesoporous silica.

**Table 4:** Representative Ionic Liquid-Enabled Delivery Systems for Biomacromolecules.

IL DDS Type	IL Function	Example System	Representative Outcome	Key Limitations
IL–polymer nanoparticle	Protein protection; oral absorption enhancement	PLGA–IL–DCA hybrid nanoparticles for oral rhGH delivery [99]	Improved GI stability and oral absorption of rhGH	Complex formulation; oral bioavailability still below injection benchmarks
IL-mediated nanovesicle (ethosome)	Protein encapsulation; membrane fluidization; transdermal permeation	Lipid-based ionic liquid ([EDMPC][Lin])-mediated ethosome for transdermal insulin delivery [101]	High insulin encapsulation efficiency, improved vesicle stability, and significantly enhanced transdermal permeation compared with conventional ethosomes	Demonstrated in vitro and ex vivo; in vivo efficacy and long-term safety remain to be established
IL-based vaccine excipient	Protein stabilization	Choline-based salt-type ILs (e.g., [Cho][Cl], [Cho][SO_4_]) for stabilization of inactivated viral antigens [102]	Improved thermal and storage stability of vaccine antigens while preserving structural integrity	Evaluated as stabilizing excipients; adjuvant (immune-enhancing) effect not established
IL-based nanoemulsion	Antigen delivery; APC activation	[Cho][Nic]-based oil-in-ionic liquid nanoemulsion for intranasal influenza split-virus vaccination [103]	Enhanced mucosal and systemic humoral and cellular immune responses via improved antigen presentation	Validated in preclinical models only
IL–DNA complex	DNA binding; nuclease protection	Choline ester-based IL complexes for DNA stabilization [104]	DNA complexation and nuclease protection; retrievable DNA	Complex stability depends on pH/additives; in vivo delivery not established
IL–siRNA complexes (topical)	Electrostatic complexation; nuclease protection; skin penetration	Ionic liquid-mediated topical delivery of siRNA for gene silencing [105]	Effective dermal delivery and target gene knockdown in an in vivo disease model	Route-limited (topical); IL-specific performance; long-term safety remains unclear

This table summarizes representative ionic liquid (IL)-enabled delivery systems for biomacromolecules, including IL functions, example formulations, reported delivery outcomes, and key limitations. Abbreviations: rhGH, recombinant human growth hormone; PLGA, poly(lactic-co-glycolic acid); DCA, deoxycholic acid; GI, gastrointestinal.

**Table 5:** Representative Ionic Liquid-Enabled Delivery Systems for Biomacromolecules across Different Administration Routes.

IL DDS Type	IL Function	Example System	Representative Outcome	Key Limitations
IL-assisted transdermal nanocarrier (Transdermal)	Skin permeation enhancement; macromolecule delivery	Ionic liquid-mediated nanovesicles for transdermal insulin delivery [123]	Enhanced transdermal transport of insulin and improved pharmacological response in preclinical models	Potential skin barrier disruption; long-term dermal safety and dose control require further evaluation
API–IL formulation (Oral)	Solubility enhancement; improved oral bioavailability	Favipiravir-based ionic liquid formulations [18]	78–125-fold increase in aqueous solubility and significantly improved oral bioavailability compared with crystalline API	Gastrointestinal tolerability and structure-dependent safety must be systematically assessed in vivo
Mixed IL system (Oral)	Extreme solubilization; rapid drug release	[Ch][Tre]–[Ch][Ger] system for tretinoin [49]	Extreme apparent content/solubility enhancement (~1.75 × 10^8^-fold vs. water solubility), rapid release (95–97% at 5 min), and increased oral exposure (Cmax, AUC)	Formulation complexity, reproducibility, and oral safety require further validation
Ionic co-aggregate based system (Injectable)	Solubilization of poorly soluble API; IV compatibility	Choline oleate ionic co-aggregates for injectable delivery of hydrophobic drugs (e.g., paclitaxel) [40]	Improved solubilization and formulation stability relevant to injectable use	Strict regulatory requirements; hemocompatibility, immunogenicity, and impurity control remain critical challenges

This table summarizes representative ionic liquid (IL)-enabled delivery systems, highlighting their primary functions, formulation outcomes, and key route-specific translational limitations.

**Table 6:** Microbial evidence linking ionic-liquid structure to antimicrobial mechanisms and Gram-type-dependent responses.

Example Focus	IL (Cation–Anion)	Bacterial Model(s)	Readout	Key Observation
Alkyl chain-dependent SAR	Imidazolium ILs with varied alkyl chain length and charge density	*E. coli*, *S. aureus*	Growth inhibition assays	Changes in alkyl chain length and cation charge density led to distinct antibacterial activities, demonstrating a clear structure–activity relationship across Gram-positive and Gram-negative bacteria [142].
Mechanism-informed activity in Gram-negative bacteria	ILs with different cation–anion combinations	*E. coli*	Antibacterial assays with mode-of-action analysis	Modulation of cation and anion structures altered antibacterial potency and was associated with distinct mode-of-action profiles in *E. coli* [143].
Gram-type susceptibility and antibiofilm effects	Long-chain cholinium-based ILs (C12–C16)	Gram-positive and Gram-negative bacteria	MIC and antibiofilm assays	Long-chain cholinium ILs showed enhanced antibacterial and antibiofilm activity, with generally higher efficacy against Gram-positive bacteria than Gram-negative strains [144].
Alkyl chain optimization and Gram-type trends	Imidazolium ILs with varying alkyl chain length and anions	*Gram-positive* and *Gram-negative bacterial panel*	MIC determination	Antimicrobial activity depended strongly on alkyl chain length, with Gram-negative bacteria typically exhibiting higher MIC values [145].
Anti-adhesive versus bactericidal behavior	Imidazolium- and pyridinium-based ILs	*Pathogenic bacteria* (*S. aureus*, *E. coli*, *P. aeruginosa*, *K. pneumoniae*)	MIC/MBC and anti-adhesion assay	Certain ILs, particularly pyridinium-based systems, showed pronounced anti-adhesive activity relative to bactericidal potency, indicating surface-associated inhibition [146].

This table summarizes representative microbial studies demonstrating how ionic-liquid structure governs antimicrobial mechanisms and Gram-type-dependent responses, based on assay-defined endpoints such as MIC, growth inhibition, and anti-adhesion or antibiofilm activity.

## Data Availability

No new data were created or analyzed in this study. Data sharing is not applicable to this article.

## References

[1] Moshikur R.M., Carrier R.L., Moniruzzaman M., Goto M. (2023). Recent Advances in Biocompatible Ionic Liquids in Drug Formulation and Delivery. Pharmaceutics.

[2] Caparica R., Julio A., Baby A.R., Araujo M.E.M., Fernandes A.S., Costa J.G., Santos de Almeida T. (2018). Choline-Amino Acid Ionic Liquids as Green Functional Excipients to Enhance Drug Solubility. Pharmaceutics.

[3] Egorova K.S., Gordeev E.G., Ananikov V.P. (2017). Biological Activity of Ionic Liquids and Their Application in Pharmaceutics and Medicine. Chem. Rev..

[4] Huang W., Yang Y., Zhao B., Liang G., Liu S., Liu X.L., Yu D.G. (2018). Fast Dissolving of Ferulic Acid via Electrospun Ternary Amorphous Composites Produced by a Coaxial Process. Pharmaceutics.

[5] Curreri A.M., Mitragotri S., Tanner E.E.L. (2021). Recent Advances in Ionic Liquids in Biomedicine. Adv Sci..

[6] Huang W., Wu X., Qi J., Zhu Q., Wu W., Lu Y., Chen Z. (2020). Ionic liquids: Green and tailor-made solvents in drug delivery. Drug Discov. Today.

[7] Gupta A., Kulkarni S., Soman S., Saha M., Kulkarni J., Rana K., Dhas N., Ayesha Farhana S., Kumar Tiyyagura P., Pandey A. (2024). Breaking barriers in cancer management: The promising role of microsphere conjugates in cancer diagnosis and therapy. Int. J. Pharm..

[8] Li W.-Z., Han W.-X., Guan L., Chen C., Zhao N., Liang F., Guo W., Fu L.-N., Yang L.-B. (2024). Hydrogel microspheres based on Bletilla striata polysaccharide as a promising cervical positioning drug delivery system. J. Drug Deliv. Sci. Technol..

[9] Yawalkar A.N., Pawar M.A., Vavia P.R. (2022). Microspheres for targeted drug delivery- A review on recent applications. J. Drug Deliv. Sci. Technol..

[10] Shukla M.K., Tiwari H., Verma R., Dong W.L., Azizov S., Kumar B., Pandey S., Kumar D. (2023). Role and Recent Advancements of Ionic Liquids in Drug Delivery Systems. Pharmaceutics.

[11] Correia D.M., Fernandes L.C., Fernandes M.M., Hermenegildo B., Meira R.M., Ribeiro C., Ribeiro S., Reguera J., Lanceros-Mendez S. (2021). Ionic Liquid-Based Materials for Biomedical Applications. Nanomaterials.

[12] Safdar R., Nawaz M., Mushtaq A., Khanh Tran T., Aziz Omar A. (2023). A bibliometric analysis for estimating the global research trends related to applications of ionic liquids in drug delivery. J. Mol. Liq..

[13] Alves M.M.S., Leandro P., Mertens H.D.T., Pereiro A.B., Archer M. (2022). Impact of Fluorinated Ionic Liquids on Human Phenylalanine Hydroxylase-A Potential Drug Delivery System. Nanomaterials.

[14] Lei Z., Chen B., Koo Y.M., MacFarlane D.R. (2017). Introduction: Ionic Liquids. Chem. Rev..

[15] Zhang R., Ahmed A., Yu B., Cong H., Shen Y. (2022). Preparation, application and development of poly(ionic liquid) microspheres. J. Mol. Liq..

[16] Abdulnabi S.A., Allami M.S. (2025). Ionic Liquids in Pharmaceutics: Biocompatibility, Physicochemical Properties, and Applications of API-ILs in Modern Drug Delivery Systems. Indones. J. Health Sci. Med..

[17] Grand View Research (2024). Ionic Liquids Market Size, Share & Trends Analysis Report by Application, by Region, and Segment Forecasts, 2024–2030.

[18] Moshikur R.M., Ali M.K., Wakabayashi R., Moniruzzaman M., Goto M. (2021). Favipiravir-Based Ionic Liquids as Potent Antiviral Drugs for Oral Delivery: Synthesis, Solubility, and Pharmacokinetic Evaluation. Mol. Pharm..

[19] Cook K., Tarnawsky K., Swinton A.J., Yang D.D., Senetra A.S., Caputo G.A., Carone B.R., Vaden T.D. (2019). Correlating Lipid Membrane Permeabilities of Imidazolium Ionic Liquids with their Cytotoxicities on Yeast, Bacterial, and Mammalian Cells. Biomolecules.

[20] Navti P.D., Pandey A., Nikam A.N., Padya B.S., Kalthur G., Koteshwara K.B., Mutalik S. (2022). Ionic Liquids Assisted Topical Drug Delivery for Permeation Enhancement: Formulation Strategies, Biomedical Applications, and Toxicological Perspective. AAPS PharmSciTech.

[21] Berton P., Shamshina J.L. (2023). Ionic Liquids as Tools to Incorporate Pharmaceutical Ingredients into Biopolymer-Based Drug Delivery Systems. Pharmaceuticals.

[22] Plechkova N.V., Seddon K.R. (2008). Applications of ionic liquids in the chemical industry. Chem. Soc. Rev..

[23] Swatloski R.P., Holbrey J.D., Rogers R.D. (2003). Ionic liquids are not always green: Hydrolysis of 1-butyl-3-methylimidazolium hexafluorophosphate. Green. Chem..

[24] Davis J.H. (2004). Task-Specific Ionic Liquids. Chem. Lett..

[25] Stoimenovski J., MacFarlane D.R., Bica K., Rogers R.D. (2010). Crystalline vs. ionic liquid salt forms of active pharmaceutical ingredients: A position paper. Pharm. Res..

[26] Hallett J.P., Welton T. (2011). Room-temperature ionic liquids: Solvents for synthesis and catalysis. 2. Chem. Rev..

[27] Ferraz R., Branco L.C., Prudencio C., Noronha J.P., Petrovski Z. (2011). Ionic liquids as active pharmaceutical ingredients. ChemMedChem.

[28] Zhuang W., Hachem K., Bokov D., Javed Ansari M., Taghvaie Nakhjiri A. (2022). Ionic liquids in pharmaceutical industry: A systematic review on applications and future perspectives. J. Mol. Liq..

[29] Handa M., Almalki W.H., Shukla R., Afzal O., Altamimi A.S.A., Beg S., Rahman M. (2022). Active pharmaceutical ingredients (APIs) in ionic liquids: An effective approach for API physiochemical parameter optimization. Drug Discov. Today.

[30] Khan O., Bhawale R., Vasave R., Mehra N.K. (2024). Ionic liquid-based formulation approaches for enhanced transmucosal drug delivery. Drug Discov. Today.

[31] Gorke J., Srienc F., Kazlauskas R. (2010). Toward advanced ionic liquids. Polar, enzyme-friendly solvents for biocatalysis. Biotechnol. Bioprocess. Eng..

[32] Jadhav N.R., Bhosale S.P., Bhosale S.S., Mali S.D., Toraskar P.B., Kadam T.S. (2021). Ionic liquids: Formulation avenues, drug delivery and therapeutic updates. J. Drug Deliv. Sci. Technol..

[33] Pillai V.V.S., Kumari P., Kolagatla S., Garcia Sakai V., Rudic S., Rodriguez B.J., Rubini M., Tych K.M., Benedetto A. (2022). Controlling Amyloid Fibril Properties Via Ionic Liquids: The Representative Case of Ethylammonium Nitrate and Tetramethylguanidinium Acetate on the Amyloidogenesis of Lysozyme. J. Phys. Chem. Lett..

[34] Maginn E.J. (2009). Molecular simulation of ionic liquids: Current status and future opportunities. J. Phys. Condens. Matter.

[35] Kuddushi M., Xu B.B., Malek N., Zhang X. (2024). Review of ionic liquid and ionogel-based biomaterials for advanced drug delivery. Adv. Colloid Interface Sci..

[36] Pedro S.N., CS R.F., Silvestre A.J.D., Freire M.G. (2020). The Role of Ionic Liquids in the Pharmaceutical Field: An Overview of Relevant Applications. Int. J. Mol. Sci..

[37] Miskiewicz A., Ceranowicz P., Szymczak M., Bartus K., Kowalczyk P. (2018). The Use of Liquids Ionic Fluids as Pharmaceutically Active Substances Helpful in Combating Nosocomial Infections Induced by Klebsiella Pneumoniae New Delhi Strain, Acinetobacter Baumannii and Enterococcus Species. Int. J. Mol. Sci..

[38] Hough W.L., Smiglak M., Rodríguez H., Swatloski R.P., Spear S.K., Daly D.T., Pernak J., Grisel J.E., Carliss R.D., Soutullo M.D. (2007). The third evolution of ionic liquids: Active pharmaceutical ingredients. New J. Chem..

[39] Li X., Ma N., Zhang L., Ling G., Zhang P. (2022). Applications of choline-based ionic liquids in drug delivery. Int. J. Pharm..

[40] Wu X., Zhu Q., Chen Z., Wu W., Lu Y., Qi J. (2021). Ionic liquids as a useful tool for tailoring active pharmaceutical ingredients. J. Control Release.

[41] Júlio A., Costa Lima S.A., Reis S., Santos de Almeida T., Fonte P. (2020). Development of ionic liquid-polymer nanoparticle hybrid systems for delivery of poorly soluble drugs. J. Drug Deliv. Sci. Technol..

[42] Onoue S., Yamada K., Sato H. (2025). Advanced oral drug delivery systems: Current challenges and emerging technologies. Acta Pharm. Sin. B.

[43] Md Moshikur R., Chowdhury M.R., Moniruzzaman M., Goto M. (2020). Biocompatible ionic liquids and their applications in pharmaceutics. Green. Chem..

[44] Keihankhadiv S., Neugebauer D. (2023). Synthesis and Characterization of Linear Copolymers Based on Pharmaceutically Functionalized Monomeric Choline Ionic Liquid for Delivery of p-Aminosalicylate. Pharmaceutics.

[45] Moshikur R.M., Ali M.K., Moniruzzaman M., Goto M. (2021). Recent advances in surface-active ionic liquid-assisted self-assembly systems for drug delivery. Curr. Opin. Colloid Interface Sci..

[46] Zhang D., Wang H.J., Cui X.M., Wang C.X. (2017). Evaluations of imidazolium ionic liquids as novel skin permeation enhancers for drug transdermal delivery. Pharm. Dev. Technol..

[47] Alves M., Vieira N.S.M., Rebelo L.P.N., Araujo J.M.M., Pereiro A.B., Archer M. (2017). Fluorinated ionic liquids for protein drug delivery systems: Investigating their impact on the structure and function of lysozyme. Int. J. Pharm..

[48] Tang J., Zhang R., Guo M., Zhou H., Zhao Y., Liu Y., Wu Y., Chen C. (2020). Gd-metallofullerenol drug delivery system mediated macrophage polarization enhances the efficiency of chemotherapy. J. Control Release.

[49] Xuan J., Wu X., Li L., Qi J., Lu X., Zhuang J. (2024). Improving oral absorption of tretinoin by ionic liquids technology. J. Drug Deliv. Sci. Technol..

[50] Verissimo N.V., Vicente F.A., de Oliveira R.C., Likozar B., Oliveira R.P.S., Pereira J.F.B. (2022). Ionic liquids as protein stabilizers for biological and biomedical applications: A review. Biotechnol. Adv..

[51] Wu J., Sahoo J.K., Li Y., Xu Q., Kaplan D.L. (2022). Challenges in delivering therapeutic peptides and proteins: A silk-based solution. J. Control Release.

[52] Lin X., Su Z., Yang Y., Zhang S. (2021). The potential of ionic liquids in biopharmaceutical engineering. Chin. J. Chem. Eng..

[53] Mirhadi E., Kesharwani P., Jha S.K., Karav S., Sahebkar A. (2024). Utilizing ionic liquids as eco-friendly and sustainable carriers for delivering nucleic acids: A review on the revolutionary advancement in nano delivery systems. Int. J. Biol. Macromol..

[54] Ossowicz-Rupniewska P., Klebeko J., Georgieva I., Apostolova S., Struk L., Todinova S., Tzoneva R.D., Guncheva M. (2024). Tuning of the Anti-Breast Cancer Activity of Betulinic Acid via Its Conversion to Ionic Liquids. Pharmaceutics.

[55] Chavda V.P., Grumezescu A.M. (2019). Nanobased Nano Drug Delivery: A Comprehensive Review. Applications of Targeted Nano Drugs and Delivery Systems.

[56] Naves L.B., Dhand C., Venugopal J.R., Rajamani L., Ramakrishna S., Almeida L. (2017). Nanotechnology for the treatment of melanoma skin cancer. Prog. Biomater..

[57] Dianzani C., Zara G.P., Maina G., Pettazzoni P., Pizzimenti S., Rossi F., Gigliotti C.L., Ciamporcero E.S., Daga M., Barrera G. (2014). Drug delivery nanoparticles in skin cancers. Biomed. Res. Int..

[58] Mahkam M., Bazmi Zeynabad F., Alizadeh E., Rahimi M., Rahimi F., Salehi R. (2021). Novel Methotrexate-Ciprofloxacin Loaded Alginate-Clay Based Nanocomposite as Anticancer and Antibacterial Co-Drug Delivery System. Adv. Pharm. Bull..

[59] Khan R.A., Mohammed H.A., Sulaiman G.M., Subaiyel A.A., Karuppaiah A., Rahman H., Makhathini S., Ramburrun P., Choonara Y.E. (2022). Molecule(s) of Interest: I. Ionic Liquids-Gateway to Newer Nanotechnology Applications: Advanced Nanobiotechnical Uses’, Current Status, Emerging Trends, Challenges, and Prospects. Int. J. Mol. Sci..

[60] Matczuk M., Timerbaev A.R., Keppler B.K., Ruzik L. (2024). Ionic liquid-mediated drug delivery: A review on progress and challenges focused on poly(ionic liquid) nanoplatforms. J. Mol. Liq..

[61] Das M., Parhi R. (2025). Nanocarriers and their integrated microneedle systems-mediated drug delivery for the treatment of moderate-severe dermatological diseases: Recent progress, applications and future perspectives. J. Drug Deliv. Sci. Technol..

[62] Kim M., Shin M., Zhao Y., Ghosh M., Son Y.O. (2024). Transformative Impact of Nanocarrier-Mediated Drug Delivery: Overcoming Biological Barriers and Expanding Therapeutic Horizons. Small Sci..

[63] Lee J.H., Yeo Y. (2015). Controlled Drug Release from Pharmaceutical Nanocarriers. Chem. Eng. Sci..

[64] Chenxi Z., Hemmat A., Thi N.H., Afrand M. (2025). Nanoparticle-enhanced drug delivery systems: An up-to-date review. J. Mol. Liq..

[65] Lu B., Zhou G., Xiao F., He Q., Zhang J. (2020). Stimuli-responsive poly(ionic liquid) nanoparticles for controlled drug delivery. J. Mater. Chem. B.

[66] Santos de Almeida T., Júlio A., Portugal Mota J., Rijo P., Reis C.P. (2017). An emerging integration between ionic liquids and nanotechnology: General uses and future prospects in drug delivery. Ther. Deliv..

[67] Ali M.K., Moshikur R.M., Wakabayashi R., Moniruzzaman M., Goto M. (2021). Biocompatible Ionic Liquid-Mediated Micelles for Enhanced Transdermal Delivery of Paclitaxel. ACS Appl. Mater. Interfaces.

[68] He Z., Alexandridis P. (2017). Ionic liquid and nanoparticle hybrid systems: Emerging applications. Adv. Colloid Interface Sci..

[69] Julio A., Caparica R., Costa Lima S.A., Fernandes A.S., Rosado C., Prazeres D.M.F., Reis S., Santos de Almeida T., Fonte P. (2019). Ionic Liquid-Polymer Nanoparticle Hybrid Systems as New Tools to Deliver Poorly Soluble Drugs. Nanomaterials.

[70] Peyvand P., Vaezi Z., Sedghi M., Dalir N., Ma’mani L., Naderi-Manesh H. (2020). Imidazolium-based ionic liquid functionalized mesoporous silica nanoparticles as a promising nano-carrier: Response surface strategy to investigate and optimize loading and release process for Lapatinib delivery. Pharm. Dev. Technol..

[71] Dasanayake G.S., Hamadani C.M., Singh G., Kumar Misra S., Vashisth P., Sharp J.S., Adhikari L., Baker G.A., Tanner E.E.L. (2024). Imidazolium-based zwitterionic liquid-modified PEG-PLGA nanoparticles as a potential intravenous drug delivery carrier. Nanoscale.

[72] Kuroda K. (2022). A simple overview of toxicity of ionic liquids and designs of biocompatible ionic liquids. New J. Chem..

[73] Xing Y., Hu Y., Zhang X., Zheng D., Ma G., Diao Y., Yue H., Wei W., Zhang S. (2025). Cationic alkyl chain length and nanoaggregate form of ionic liquids dominate biocompatibility and toxicity. Nat. Commun..

[74] Hu Y., Xing Y., Yue H., Chen T., Diao Y., Wei W., Zhang S. (2023). Ionic liquids revolutionizing biomedicine: Recent advances and emerging opportunities. Chem. Soc. Rev..

[75] Costa C., Padrela L. (2025). Progress on drug nanoparticle manufacturing: Exploring the adaptability of batch bottom-up approaches to continuous manufacturing. J. Drug Deliv. Sci. Technol..

[76] Lin H., Leng J., Fan P., Xu Z., Ruan G. (2023). Scalable production of microscopic particles for biological delivery. Mater. Adv..

[77] Goncalves Pereira I.L., Ziulkoski A.L., Zepon K.M., Kanis L.A., Schrekker H.S. (2026). Ionic Liquids in Pharmaceuticals: A Scoping Review of Formulation Strategies. ACS Omega.

[78] Jain A., Shakya A.K., Prajapati S.K., Eldesoqui M., Mody N., Jain S.K., Naik R.R., Patil U.K. (2024). An insight into pharmaceutical challenges with ionic liquids: Where do we stand in transdermal delivery?. Front. Bioeng. Biotechnol..

[79] Sun J., Yang Z., Teng L. (2020). Nanotechnology and Microtechnology in Drug Delivery Systems. Dose Response.

[80] Stevenson C.L., Santini J.T., Langer R. (2012). Reservoir-based drug delivery systems utilizing microtechnology. Adv. Drug Deliv. Rev..

[81] Luo R., Xu H., Lin Q., Chi J., Liu T., Jin B., Ou J., Xu Z., Peng T., Quan G. (2024). Emerging Trends in Dissolving-Microneedle Technology for Antimicrobial Skin-Infection Therapies. Pharmaceutics.

[82] Salabat A., Moniruzzaman M.G.M. (2021). Ionic Liquid Assisted Microemulsions for Drug Delivery. Application of Ionic Liquids in Drug Delivery.

[83] Zhang T., Sun B., Guo J., Wang M., Cui H., Mao H., Wang B., Yan F. (2020). Active pharmaceutical ingredient poly(ionic liquid)-based microneedles for the treatment of skin acne infection. Acta Biomater..

[84] Zhang Q., Zhang Z., Zou X., Liu Z., Li Q., Zhou J., Gao S., Xu H., Guo J., Yan F. (2023). Nitric oxide-releasing poly(ionic liquid)-based microneedle for subcutaneous fungal infection treatment. Biomater. Sci..

[85] Zhu Y., Zhou Y., Ma X., Duan Z., Xu H., Li Y., Kong Y., Yang L., Xin X. (2025). Topical Therapy in Psoriasis: Clinical Benefits, Advances in Novel Drug Delivery Strategies, and Gene Therapy Regimen. Pharmaceutics.

[86] Rao V.G., Mandal S., Ghosh S., Banerjee C., Sarkar N. (2012). Ionic liquid-in-oil microemulsions composed of double chain surface active ionic liquid as a surfactant: Temperature dependent solvent and rotational relaxation dynamics of coumarin-153 in [Py][TF2N]/[C4mim][AOT]/benzene microemulsions. J. Phys. Chem. B.

[87] Kuchlyan J., Kundu N., Sarkar N. (2016). Ionic liquids in microemulsions: Formulation and characterization. Curr. Opin. Colloid Interface Sci..

[88] Kogan A., Garti N. (2006). Microemulsions as transdermal drug delivery vehicles. Adv. Colloid Interface Sci..

[89] Moniruzzaman M., Tamura M., Tahara Y., Kamiya N., Goto M. (2010). Ionic liquid-in-oil microemulsion as a potential carrier of sparingly soluble drug: Characterization and cytotoxicity evaluation. Int. J. Pharm..

[90] Rao V.G., Banerjee C., Ghosh S., Mandal S., Kuchlyan J., Sarkar N. (2013). A step toward the development of high-temperature stable ionic liquid-in-oil microemulsions containing double-chain anionic surface active ionic liquid. J. Phys. Chem. B.

[91] Lu B., Liu T., Wang H., Wu C., Chen H., Liu Z., Zhang J. (2022). Ionic liquid transdermal delivery system: Progress, prospects, and challenges. J. Mol. Liq..

[92] Goindi S., Arora P., Kumar N., Puri A. (2014). Development of novel ionic liquid-based microemulsion formulation for dermal delivery of 5-Fluorouracil. AAPS PharmSciTech.

[93] Avcil M., Celik A. (2021). Microneedles in Drug Delivery: Progress and Challenges. Micromachines.

[94] Aldawood F.K., Andar A., Desai S. (2021). A Comprehensive Review of Microneedles: Types, Materials, Processes, Characterizations and Applications. Polymers.

[95] Correia A., Agostinho Cordeiro M., Mendes M., Marques Ribeiro M., Mascarenhas-Melo F., Vitorino C. (2026). Additive manufacturing of microneedles: A quality by design approach to clinical translation. Int. J. Pharm..

[96] Nadda R., Singh P.K., Das D.B. (2024). Revolutionizing microneedle array fabrication using additive manufacturing technologies: Potential applications and clinical translation. J. Drug Deliv. Sci. Technol..

[97] Yang R., Tee X.Y., Poornachary S.K., Simone E., Chow P.S. (2025). Influence of Processing and Stabilizer Selection on Microstructure, Stability and Rheology of Emulsion-Based Semisolid Formulations. Pharmaceutics.

[98] Vieira N.S.M., Castro P.J., Marques D.F., Araujo J.M.M., Pereiro A.B. (2020). Tailor-Made Fluorinated Ionic Liquids for Protein Delivery. Nanomaterials.

[99] Zhou J., Li S., Zhang J., Luo F., Sun Y., Guan M., Ma H., Liu Q. (2023). Ionic liquid combined with bile acid pathway for oral delivery of rhGH. J. Drug Deliv. Sci. Technol..

[100] Badr-Eldin E.H. (2021). Potential Application of Ionic Liquids in Pharmaceutical Dosage Forms for Enhanced Solubility, Permeation and Bioavailability. Pharmaceuticals.

[101] Nabila F.H., Islam R., Shimul I.M., Moniruzzaman M., Wakabayashi R., Kamiya N., Goto M. (2024). Ionic liquid-mediated ethosome for transdermal delivery of insulin. Chem. Commun..

[102] Lin X., Yang Y., Li S., Song Y., Ma G., Su Z., Zhang S. (2019). Unique stabilizing mechanism provided by biocompatible choline-based ionic liquids for inhibiting dissociation of inactivated foot-and-mouth disease virus particles. RSC Adv..

[103] Lin X., Sheng Y., Zhang X., Li Z., Yang Y., Wu J., Su Z., Ma G., Zhang S. (2022). Oil-in-ionic liquid nanoemulsion-based intranasal delivery system for influenza split-virus vaccine. J. Control Release.

[104] Sagitha P., Dhandapani H., Tayalia P. (2023). Choline ester based ionic liquid: A multi-functional system to enhance nucleic acid stability, drug solubilization and cell penetration. Int. J. Biol. Macromol..

[105] Dharamdasani V., Mandal A., Qi Q.M., Suzuki I., Bentley M., Mitragotri S. (2020). Topical delivery of siRNA into skin using ionic liquids. J. Control Release.

[106] Mukesh C., Mondal D., Sharma M., Prasad K. (2013). Rapid dissolution of DNA in a novel bio-based ionic liquid with long-term structural and chemical stability: Successful recycling of the ionic liquid for reuse in the process. Chem. Commun..

[107] Egorova K.S., Posvyatenko A.V., Larin S.S., Ananikov V.P. (2021). Ionic liquids: Prospects for nucleic acid handling and delivery. Nucleic Acids Res..

[108] Pedro A.Q., Pereira P., Quental M.J., Carvalho A.P., Santos S.M., Queiroz J.A., Sousa F., Freire M.G. (2018). Cholinium-based Good’s buffers ionic liquids as remarkable stabilizers and recyclable preservation media for recombinant small RNAs. ACS Sustain. Chem. Eng..

[109] Shukla S.K., Mikkola J.P. (2020). Use of Ionic Liquids in Protein and DNA Chemistry. Front. Chem..

[110] Fujita K., Ohno H. (2012). Stable G-quadruplex structure in a hydrated ion pair: Cholinium cation and dihydrogen phosphate anion. Chem. Commun..

[111] Coffman R.L., Sher A., Seder R.A. (2010). Vaccine adjuvants: Putting innate immunity to work. Immunity.

[112] Kumru O.S., Joshi S.B., Smith D.E., Middaugh C.R., Prusik T., Volkin D.B. (2014). Vaccine instability in the cold chain: Mechanisms, analysis and formulation strategies. Biologicals.

[113] Akkineni S., Rawas-Qalaji M., Kouzi S.A., Chbib C., Uddin M.N. (2025). Exploring the Biological Activities of Ionic Liquids and Their Potential to Develop Novel Vaccine Adjuvants. Vaccines.

[114] Zhuo Y., Cheng H.L., Zhao Y.G., Cui H.R. (2024). Ionic Liquids in Pharmaceutical and Biomedical Applications: A Review. Pharmaceutics.

[115] Losada-Barreiro S., Celik S., Sezgin-Bayindir Z., Bravo-Fernandez S., Bravo-Diaz C. (2024). Carrier Systems for Advanced Drug Delivery: Improving Drug Solubility/Bioavailability and Administration Routes. Pharmaceutics.

[116] Liu C., Chen B., Shi W., Huang W., Qian H. (2022). Ionic Liquids for Enhanced Drug Delivery: Recent Progress and Prevailing Challenges. Mol. Pharm..

[117] Dobler D., Schmidts T., Klingenhofer I., Runkel F. (2013). Ionic liquids as ingredients in topical drug delivery systems. Int. J. Pharm..

[118] Beaven E., Kumar R., An J.M., Mendoza H., Sutradhar S.C., Choi W., Narayan M., Lee Y.K., Nurunnabi M. (2024). Potentials of ionic liquids to overcome physical and biological barriers. Adv. Drug Deliv. Rev..

[119] Islam M.R., Chowdhury M.R., Wakabayashi R., Tahara Y., Kamiya N., Moniruzzaman M., Goto M. (2020). Choline and amino acid based biocompatible ionic liquid mediated transdermal delivery of the sparingly soluble drug acyclovir. Int. J. Pharm..

[120] Paraskevopoulos G., Fandrei F., Kumar Pratihast A., Paraskevopoulou A., Panoutsopoulou E., Opalka L., Singh Mithu V., Huster D., Vavrova K. (2024). Effects of imidazolium ionic liquids on skin barrier lipids—Perspectives for drug delivery. J. Colloid Interface Sci..

[121] Sidat Z., Marimuthu T., Kumar P., du Toit L.C., Kondiah P.P.D., Choonara Y.E., Pillay V. (2019). Ionic Liquids as Potential and Synergistic Permeation Enhancers for Transdermal Drug Delivery. Pharmaceutics.

[122] Li Y., Yu Q., Lu Y., He H., Qi J., Tai Z., Chen Z., Zhu Q., Wu W. (2024). Enhanced transdermal delivery of insulin by choline-based ionic liquids. Int. J. Pharm..

[123] Nabila F.H., Islam R., Yamin L., Yoshirou K., Wakabayashi R., Kamiya N., Moniruzzaman M., Goto M. (2025). Transdermal Insulin Delivery Using Ionic Liquid-Mediated Nanovesicles for Diabetes Treatment. ACS Biomater. Sci. Eng..

[124] Lou J., Duan H., Qin Q., Teng Z., Gan F., Zhou X., Zhou X. (2023). Advances in Oral Drug Delivery Systems: Challenges and Opportunities. Pharmaceutics.

[125] Spiljak B., Somogyi Skoc M., Rezic Mestrovic I., Basic K., Bando I., Sutej I. (2025). Targeting the Oral Mucosa: Emerging Drug Delivery Platforms and the Therapeutic Potential of Glycosaminoglycans. Pharmaceutics.

[126] Samanthula K.S., Bairi A.G., Kothapally D. (2025). Transbuccal Drug Delivery Systems: A Comprehensive Review of Recent Approaches. J. Young Pharm..

[127] Shamshina J.L., Barber P.S., Rogers R.D. (2013). Ionic liquids in drug delivery. Expert Opin. Drug Deliv..

[128] Chu J.N., Traverso G. (2022). Foundations of gastrointestinal-based drug delivery and future developments. Nat. Rev. Gastroenterol. Hepatol..

[129] Hua S. (2020). Advances in Oral Drug Delivery for Regional Targeting in the Gastrointestinal Tract—Influence of Physiological, Pathophysiological and Pharmaceutical Factors. Front. Pharmacol..

[130] Li S., Wu T., Wu J., Chen W., Zhang D. (2024). Recognizing the biological barriers and pathophysiological characteristics of the gastrointestinal tract for the design and application of nanotherapeutics. Drug Deliv..

[131] Vinarov Z., Abdallah M., Agundez J.A.G., Allegaert K., Basit A.W., Braeckmans M., Ceulemans J., Corsetti M., Griffin B.T., Grimm M. (2021). Impact of gastrointestinal tract variability on oral drug absorption and pharmacokinetics: An UNGAP review. Eur. J. Pharm. Sci..

[132] Allen T.M., Cullis P.R. (2004). Drug delivery systems: Entering the mainstream. Science.

[133] Jin J.F., Zhu L.L., Chen M., Xu H.M., Wang H.F., Feng X.Q., Zhu X.P., Zhou Q. (2015). The optimal choice of medication administration route regarding intravenous, intramuscular, and subcutaneous injection. Patient Prefer. Adherence.

[134] Kim H., Park H., Lee S.J. (2017). Effective method for drug injection into subcutaneous tissue. Sci. Rep..

[135] Nabila F.H., Moniruzzaman M., Goto M. (2025). Ionic liquid-based transdermal drug delivery systems for biopharmaceuticals. Chem. Commun..

[136] Guo H.Y., Cao B., Deng G., Hao X.L., Wu F.G., Yu Z.W. (2019). Effect of Imidazolium-Based Ionic Liquids on the Structure and Phase Behavior of Palmitoyl-oleoyl-phosphatidylethanolamine. J. Phys. Chem. B.

[137] Kaur N., Mithu V.S., Kumar S. (2024). A review on (eco)toxicity of ionic liquids and their interaction with phospholipid membranes. J. Mol. Liq..

[138] Hossain M.I., Shams A.B., Das Gupta S., Blanchard G.J., Mobasheri A., Hoque Apu E. (2023). The Potential Role of Ionic Liquid as a Multifunctional Dental Biomaterial. Biomedicines.

[139] Mikuni-Mester P., Robben C., Witte A.K., Linke K., Ehling-Schulz M., Rossmanith P., Grunert T. (2024). Antimicrobial Ionic Liquids: Ante-Mortem Mechanisms of Pathogenic EPEC and MRSA Examined by FTIR Spectroscopy. Int. J. Mol. Sci..

[140] Fallah Z., Zare E.N., Khan M.A., Iftekhar S., Ghomi M., Sharifi E., Tajbakhsh M., Nikfarjam N., Makvandi P., Lichtfouse E. (2021). Ionic liquid-based antimicrobial materials for water treatment, air filtration, food packaging and anticorrosion coatings. Adv. Colloid Interface Sci..

[141] Novello E., Scalzo G., D’Agata G., Raucci M.G., Ambrosio L., Soriente A., Tomasello B., Restuccia C., Parafati L., Consoli G.M.L. (2024). Synthesis, Characterisation, and In Vitro Evaluation of Biocompatibility, Antibacterial and Antitumor Activity of Imidazolium Ionic Liquids. Pharmaceutics.

[142] Zheng Z., Xu Q., Guo J., Qin J., Mao H., Wang B., Yan F. (2016). Structure-Antibacterial Activity Relationships of Imidazolium-Type Ionic Liquid Monomers, Poly(ionic liquids) and Poly(ionic liquid) Membranes: Effect of Alkyl Chain Length and Cations. ACS Appl. Mater. Interfaces.

[143] Fernandes M.M., Carvalho E.O., Correia D.M., Esperanca J., Padrao J., Ivanova K., Hoyo J., Tzanov T., Lanceros-Mendez S. (2022). Ionic Liquids as Biocompatible Antibacterial Agents: A Case Study on Structure-Related Bioactivity on Escherichia coli. ACS Appl. Bio Mater..

[144] Garcia M.T., Bautista E., de la Fuente A., Perez L. (2023). Cholinium-Based Ionic Liquids as Promising Antimicrobial Agents in Pharmaceutical Applications: Surface Activity, Antibacterial Activity and Ecotoxicological Profile. Pharmaceutics.

[145] Palkowski L., Karolak M., Skrzypczak A., Wojcieszak M., Walkiewicz F., Podemski J., Jaroch K., Bojko B., Materna K., Krysinski J. (2022). Antimicrobial and Cytotoxic Activity of Novel Imidazolium-Based Ionic Liquids. Molecules.

[146] Anvari S., Hajfarajollah H., Mokhtarani B., Enayati M., Sharifi A., Mirzaei M. (2016). Antibacterial and anti-adhesive properties of ionic liquids with various cationic and anionic heads toward pathogenic bacteria. J. Mol. Liq..

[147] Pendleton J.N., Gilmore B.F. (2015). The antimicrobial potential of ionic liquids: A source of chemical diversity for infection and biofilm control. Int. J. Antimicrob. Agents.

[148] Costa F.M.S., Saraiva M.L.M.F.S., Passos M.L.C. (2022). Ionic liquids and organic salts with antimicrobial activity as a strategy against resistant microorganisms. J. Mol. Liq..

[149] Su Q., He X., Wang S., Song H., Dong J. (2024). Choline-amino acid-based polyionic liquids and ionogels for antimicrobial infections. Mater. Today Commun..

[150] Ferraz R., Teixeira V., Rodrigues D., Fernandes R., Prudêncio C., Noronha J.P., Petrovski Ž., Branco L.C. (2014). Antibacterial activity of Ionic Liquids based on ampicillin against resistant bacteria. RSC Adv..

[151] Florio W., Rizzato C., Becherini S., Guazzelli L., D’Andrea F., Lupetti A. (2020). Synergistic activity between colistin and the ionic liquids 1-methyl-3-dodecylimidazolium bromide, 1-dodecyl-1-methylpyrrolidinium bromide, or 1-dodecyl-1-methylpiperidinium bromide against Gram-negative bacteria. J. Glob. Antimicrob. Resist..

[152] Nikfarjam N., Ghomi M., Agarwal T., Hassanpour M., Sharifi E., Khorsandi D., Ali Khan M., Rossi F., Rossetti A., Nazarzadeh Zare E. (2021). Antimicrobial Ionic Liquid-Based Materials for Biomedical Applications. Adv. Funct. Mater..

[153] Abdollahi M., Andalib S., Ghorbani R., Afshar D., Gholinejad M., Abdollahi H., Akbari A., Nikfarjam N. (2024). Polydopamine contained hydrogel nanocomposites with combined antimicrobial and antioxidant properties for accelerated wound healing. Int. J. Biol. Macromol..

[154] Michalski J., Odrzygóźdź C., Mester P., Narożna D., Cłapa T. (2023). Defeat undefeatable: Ionic liquids as novel antimicrobial agents. J. Mol. Liq..

[155] Gao C., Qiu H., Chen J. (2025). Ionic liquid-based composites: Multifunctional antibacterial platform. Fundam. Res..

[156] Sivapragasam M., Moniruzzaman M., Goto M. (2020). An Overview on the Toxicological Properties of Ionic Liquids toward Microorganisms. Biotechnol. J..

[157] Chen X., Li Z., Yang C., Yang D. (2024). Ionic liquids as the effective technology for enhancing transdermal drug delivery: Design principles, roles, mechanisms, and future challenges. Asian J. Pharm. Sci..

[158] Docherty K.M., Kulpa J.C.F. (2005). Toxicity and antimicrobial activity of imidazolium and pyridinium ionic liquids. Green. Chem..

[159] Simoes M., Pereira A.R., Simoes L.C., Cagide F., Borges F. (2021). Biofilm control by ionic liquids. Drug Discov. Today.

[160] Song X., Tian R., Liu K. (2023). Recent advances in the application of ionic liquids in antimicrobial material for air disinfection and sterilization. Front. Cell Infect. Microbiol..

[161] Hajfarajollah H., Mokhtarani B., Noghabi K.A., Sharifi A., Mirzaei M. (2014). Antibacterial and antiadhesive properties of butyl-methylimidazolium ionic liquids toward pathogenic bacteria. RSC Adv..

[162] Cho C.W., Pham T.P.T., Zhao Y., Stolte S., Yun Y.S. (2021). Review of the toxic effects of ionic liquids. Sci. Total Environ..

[163] Ventura S.P., de Barros R.L., Sintra T., Soares C.M., Lima A.S., Coutinho J.A. (2012). Simple screening method to identify toxic/non-toxic ionic liquids: Agar diffusion test adaptation. Ecotoxicol. Environ. Saf..

[164] Kaur N., Kumar S., Shiksha, Gahlay G.K., Mithu V.S. (2021). Cytotoxicity and Membrane Permeability of Double-Chained 1,3-Dialkylimidazolium Cations in Ionic Liquids. J. Phys. Chem. B.

[165] Yang D.D., Paterna N.J., Senetra A.S., Casey K.R., Trieu P.D., Caputo G.A., Vaden T.D., Carone B.R. (2021). Synergistic interactions of ionic liquids and antimicrobials improve drug efficacy. iScience.

[166] Kumari P., Pillai V.V.S., Benedetto A. (2020). Mechanisms of action of ionic liquids on living cells: The state of the art. Biophys. Rev..

[167] Ferraz R., Silva D., Dias A.R., Dias V., Santos M.M., Pinheiro L., Prudencio C., Noronha J.P., Petrovski Z., Branco L.C. (2020). Synthesis and Antibacterial Activity of Ionic Liquids and Organic Salts Based on Penicillin G and Amoxicillin hydrolysate Derivatives against Resistant Bacteria. Pharmaceutics.

[168] Duman A.N., Ozturk I., Tuncel A., Ocakoglu K., Colak S.G., Hosgor-Limoncu M., Yurt F. (2019). Synthesis of new water-soluble ionic liquids and their antibacterial profile against gram-positive and gram-negative bacteria. Heliyon.

[169] Grewal J., Khare S.K., Drewniak L., Pranaw K. (2022). Recent perspectives on microbial and ionic liquid interactions with implications for biorefineries. J. Mol. Liq..

[170] Bae E., Beil S., Konig M., Stolte S., Escher B.I., Markiewicz M. (2024). The mode of toxic action of ionic liquids: Narrowing down possibilities using high-throughput, in vitro cell-based bioassays. Environ. Int..

[171] Coleman D., Gathergood N. (2010). Biodegradation studies of ionic liquids. Chem. Soc. Rev..

[172] Pham T.P., Cho C.W., Yun Y.S. (2010). Environmental fate and toxicity of ionic liquids: A review. Water Res..

[173] Flieger J., Flieger M. (2020). Ionic Liquids Toxicity-Benefits and Threats. Int. J. Mol. Sci..

